# The science and art of testing in ice hockey: a systematic review of twenty years of research

**DOI:** 10.3389/fspor.2023.1252093

**Published:** 2023-09-28

**Authors:** Michael Bournival, Gaëtan Martini, François Trudeau, Jean Lemoyne

**Affiliations:** ^1^ Laboratoire de recherche sur le hockey UQTR, Université du Québec à Trois-Rivières, Trois-Rivières, QC, Canada; ^2^ Department of Human Kinetics, Université du Québec à Trois-Rivières, Trois-Rivières, QC, Canada

**Keywords:** performance assessment, on-ice test, skating, aerobic capacity, acceleration, speed, change of direction, repeated sprint ability

## Abstract

**Introduction:**

Ice hockey is a complex sport requiring multiple athletic and technical attributes. Considering the variety of tests developed, on-ice testing protocols have been created to measure the physiological and mechanical attributes associated with performance. To our knowledge, a lack of technical resources exists to help stakeholders opt for on-ice protocols from among those developed. It becomes crucial for researchers and practitioners to select relevant and context-specific procedures. This systematic review of the literature outlines an inventory of the on-ice tests that have been used in the domain of ice hockey research over the last twenty years, and summarize protocols mostly used in major athletic components.

**Methods:**

A search was performed on three databases (PubMed, SPORTDiscus and Scopus) by following the PRISMA guidelines. Specific keywords were selected to find publications using on-ice testing protocols in the methodology. Four aspects of athletic attributes were used to categorize the protocols: aerobic capacity, acceleration-speed, agility-change of direction and ability to repeat skating sprints. Analyses were conducted regarding four categories of observations: population under study, on-ice reported test(s), outcomes measures and main findings.

**Results:**

A total of 107 articles were included, resulting in 55 on-ice tests related to the on-ice assessments of four major athletic components: aerobic capacity (*n* = 7), acceleration-speed (*n* = 6), agility and change of direction (*n* = 23) and repeated skating sprint ability (*n* = 19). Testing in male and older cohorts (≥16 years old) predominates, with a primary focus on the competitive amateur level. The selected tests were mainly designed for assessing on-ice physiological responses and fitness (*n* = 38), talent identification-team selection (*n* = 19), efficiency of interventions (*n* = 17) and validation purposes (*n* = 16).

**Conclusion:**

A prevalence of on-ice skating tests to assess the ability to repeat intense efforts, agility, acceleration and speed components exists, which are relevant and linked to match requirement. The wealth of on-ice tests used in the literature reflects the need to adapt the on-ice evaluation process to the population, constraints, and goals. This review is a valid toolbox and can benefit for researchers and practitioners interested in testing hockey players from different levels, with a variety of aims and needs, by helping them to select the relevant procedures to their environment and practice context.

## 1. Introduction

Ice hockey is a team sport that consists of multiple technical tasks such as skating, sliding, shooting and body checking and is organized with phases of play interspersed with passive recoveries (1). As an intermittent team sport, it requires attributes of acceleration, speed, power, endurance and the ability to repeat short and intense efforts (2). The physiological demands of modern ice hockey have increased over the last decades (1, 3, 4). Performance assessment has become a field of expertise that is crucial for researchers and practitioners (e.g., coaches, strength and conditioning coaches, scouts, program directors), who need to be aware of the mechanisms that predispose hockey players to perform in key situations (5). In this regard, several areas of interest are being studied: talent identification, team selection processes, performance analysis, and monitoring individual progress after strength and conditioning training (6). Conducting testing protocols are useful to determine potential and capacity to perform as well as readiness for competition in ice hockey (7). By assessing athletes and allowing a better understanding of determinants of the performance, the process of optimizing these physical attributes becomes part of player development (2, 8). As an example, it has been shown that athletes' on-ice performances can be improved by developing their athletic capacities with strength and conditioning (9, 10). However, further research is still needed to provide clear scientific evidence that supports the associations between functional fitness, on-ice testing protocols and game performance in real settings (11).

### 1.1. Testing in ice hockey: from strength and conditioning room to real-game settings

Performance in ice hockey is assessed from three main scientific perspectives: off-ice fitness or athletic capacities (12), on-ice specific fitness attributes (13, 14) and on-ice game performance (15, 16). Although previous researchers have studied functional fitness evaluation for both the youth (17, 18) and professional levels (19, 20), this approach is essential for adapting strength and conditioning training programs to an athlete's needs (2). Off-ice assessment methods commonly used (i.e., jumps, acceleration, speed, change of direction, upper and lower-body strength, shuttle run, balance), are useful for establishing an athlete's profile in order to monitor their progress over an entire season (7, 21). There is an extensive description of the testing batteries used, and their role in reaching the high levels of athlete development is well defined (22). The most common one is the NHL Draft Combine (23), which consists of a group of multiple off-ice tests. In this regard, they were shown to have weak predictive validity, especially since they may be less specific to the requirements of on-ice demands (24).

To counteract the limitations of off-ice tests, scientific interest has focused on on-ice fitness specifically in recent decades. The first on-ice testing protocols were conducted over twenty years ago with an emphasis on aerobic capacity, acceleration, speed and change of direction (25, 26, 27). On-ice performance can be assessed in multiple ways, from the empirical evaluation of on-ice skating test times with stopwatches or photo-electric cells (18, 28), modern technologies (14, 29, 30) and biomechanical and kinematics pattern movements analysis (31, 32, 33). Finally, diverse approaches allow for *in situ* assessment, where it becomes possible to measure and track players' instantaneous on-ice performance during in-game situations. Previously analyzed with traditional methods (e.g., video analysis or qualitative observation grids) (34, 35), technological improvements have promoted the accuracy and reliability of on-ice performance assessment during game situations (e.g., accelerometry, local positioning systems or automated analysis software) (36, 37, 38).

In summary, we can conclude that two performance-assessment approaches coexist in the domain of ice hockey: “off-ice” fitness tests and specific “on-ice” tests. As mentioned previously, given that off-ice tests may not be specific enough to the requirements of ice hockey, increased attention to on-ice assessment seems to offer the path to a better understanding of the mechanisms underlying performance in ice hockey (39). In this regard, systematic reviews focused on longitudinal off-ice fitness, physiological parameters and on-ice evaluation (40) as well as on-ice performance testing with an emphasis on straight sprint acceleration and speed (41). Nevertheless, many challenges remain when researchers attempt to establish associations between attributes tested on the ice and how these attributes translate into real performance in competition settings (42).

### 1.2. Objectives: bridging the gap between research and application

When seeking the most reliable and valid options for testing hockey players, researchers face multiple options regarding the best ways to assess the attributes needed to excel. Regarding a major specificity of this sport, there is currently no exhaustive literature review, to our knowledge, of on-ice test protocols used with specific populations and different levels of expertise. From this perspective, bridging the gap between science and its application in practical settings (e.g., less controlled environments) is relevant for both researchers and practitioners. For researchers, an in-depth knowledge of the methods used to assess ice-hockey attributes, with regard to strong external validity, is valuable in their search to replicate research designs specific to the populations studied. More practically, a complete repertoire of on-ice testing protocols is relevant for ice hockey and strength and conditioning coaches as well as federations because it allows them to select methods that are appropriate and adapted to their players' characteristics (e.g., sex, age, level of play, etc.). Accordingly, the aim of this systematic review is threefold: (1) to present an inventory of the on-ice testing protocols most frequently used to assess physiological and physical attributes relating to ice hockey; (2) to provide a critical overview of the characteristics used in this research including population under study (playing level, sex, age group), study design; validation; and outcomes measures, and (3) to propose recommendations for research-practitioners on the methods that were used in their areas of interest. These recommendations will serve as a framework for designing replicable designs (e.g., in research) and/or implementing testing sessions adapted to stakeholders' purposes (e.g., fitness testing, team selection, assessment goals, required equipment, etc.).

## 2. Methods

### 2.1. Identifying the research question

The systematic review process is a suitable and appropriate method to quantify several studies with different designs, establish links between them and synthesize them (43). This type of design allows researchers to answer questions such as “*How many on-ice tests are mentioned in scientific research that aim to assess the physical, technical and physiological qualities of hockey players*?” Next, it describes the population, type of study design and observed associations of the different tests in order to identify current contributions and related scientific shortcomings in the field of ice hockey. As mentioned earlier, the main objective of this review is to inform researchers of what has been done in on-ice performance testing and provide measurement tools for practitioners to assess hockey players.

### 2.2. Finding relevant studies

**Figure 1:**
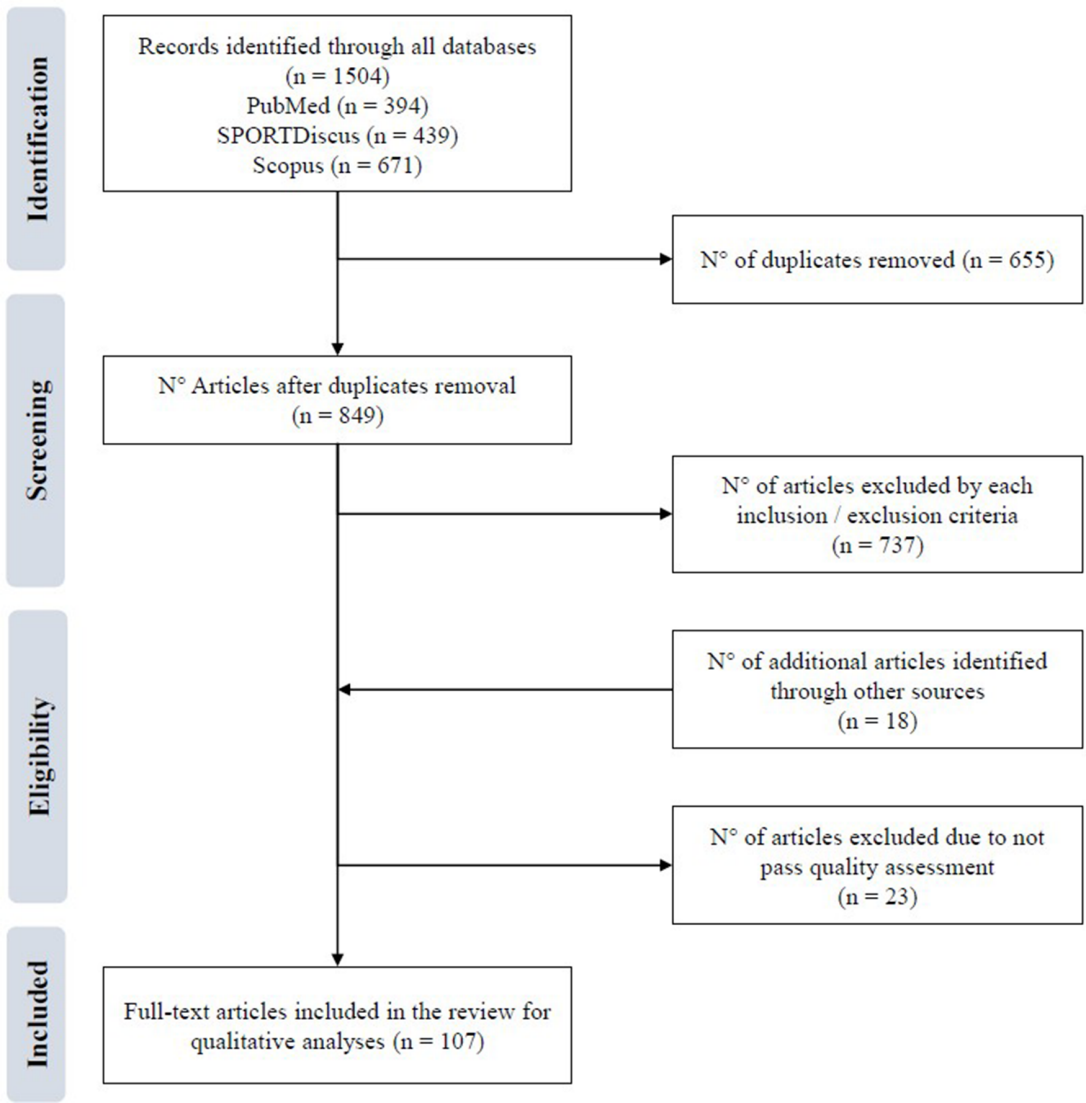
Stages of systematic review-PRISMA to identify on-ice tests in ice hockey.

Article identification and selection was done in accordance with PRISMA guidelines (44), as illustrated in Figure 1. An initial search in three main sport science databases (PubMed, SPORTDiscus and Scopus) was performed on August 2022 using the terms *ice hockey* and test* as keywords for all databases searched. Searches were limited to articles in English published since January 2000. We justify this time frame for many reasons. Firstly, the introduction of professional (NHL) players at the 1998 Nagano Olympics contributed to the globalization of ice hockey, which resulted in an increase of scholars' interest towards the science of ice hockey. The profile of modern ice hockey players also changes over the years. For example, an article published by Triplett and colleagues (45) showed that National Collegiate Athletic Association hockey players morphology has changed over the last decades. This suggest that the level of athleticism that is needed to excel in ice hockey might be different than it was in the late 1990's. Some other factors, such as rule changes (e.g., removing the blue lines, introducing 3 vs. 3 overtimes, etc.) and the emergence of multiple junior-prospects tournaments also contributed to make ice hockey evolve in terms of the required attributes to attain the highest standards. A final argument that could be used is that, from the 2000 s to the present, we have seen an acceleration in the rate of ice hockey publications (46), explaining our decision of a “2000 to 2023” time frame.

Since then, speed and agility have become an important aspect of the game. To obtain specific information regarding the validity of the selected tests, we looked for the original study without noting year of publication (between 1950 and 1999). Next, we added articles published from August 2022 to May 2023 as well as articles which were not in the databases mentioned above but which corresponded to the inclusion criteria. Endnote (Clarivate, London, United Kingdom) was used and, after all titles of the three databases were uploaded, the software automatically identified and removed duplicates. Relevant articles were then screened for eligibility after reading the title and abstract of each remaining record, following the same manual PICOS procedure strategy (Population, Intervention, Comparison, Outcomes and Study design) as for the database search and inclusion criteria (47).

### 2.3. Selecting relevant studies

Two researchers (MB and GM) independently screened the title and abstract for each selected record by applying the PICOS framework. Inclusion criteria were: (1) male and/or female ice hockey players; (2) any articles containing at least one on-ice hockey test; and (3) ice hockey on-ice test that evaluates aerobic capacity, speed, agility or change of direction and ability to repeat sprints. Exclusion criteria for eliminating irrelevant records were: (1) article was not written in English; (2) test was not performed on the ice; (3) testing procedure was not clear (e.g., skating distances or test explanations); and (4) there were no test assessing attributes other than those mentioned above (e.g., technical on-ice test). After completing these steps, the researchers read each eligible article in full to narrow the list to all relevant studies that answered the research question. If the evaluating authors disagreed about the inclusion of an article, the decision was made by a third researcher (JL), also after reading.

### 2.4. Classification of information

Based on ice hockey game performance analysis (1, 4, 48), we identified four categories of physiological and physical attributes associated with the sport requirements: (1) aerobic capacity, (2) skating acceleration and speed, (3) change of direction (CoD) and agility, and (4) Ability to repeat sprints. Aerobic capacity was defined based on physiological considerations (3, 48) and refers to the use of an incremental protocol where objective measures (e.g., skating speed, distance) increase are observed throughout the test, while subjective parameters are following the same path (e.g., heart rate, intensity, blood lactate) until exhaustion. In speed assessment, a short maximum effort is exerted once in a linear or circular fashion (28, 41). Agility tests refer to efficiency in executing preplanned changes of direction such as tight turns, braking, crossovers and transitions from one skating technique to another over a short distance (26, 49, 50). As shown by Novak and colleagues (50), the transfer from off-ice agility to on-ice skating agility seems plausible in terms of trainability among cohorts of elite under16 (U16) Czech players. A fourth component, which is repeated skating sprint ability (RSSA) is the capacity to reproduce intense or maximal short duration efforts interspersed with brief recoveries (51). However, this ability to repeat such intense efforts involves both aerobic (e.g., high number of repetitions, 60 s or less brief and partial recovery) and anaerobic (e.g., short duration intense efforts less than 10 s) energy systems at the same time. RSSA is therefore considered an important attribute of the sport in ice hockey (48, 52). Furthermore, we are aware that hockey skills are a key component of the toolbox a hockey player uses to excel during a game. Thus, most ice hockey federations have developed testing batteries to test hockey players' skills at different stages of their development. The International Ice Hockey Federation (IIHF) tests for talent identification and Hockey Canada's National Skills Standards and Testing Program are good examples of such materials (53, 54). However, we decided to exclude these kinds of protocols, since the aim of this review is to identify tests used in a research context focused on on-ice physiological and athletic testing. For each category of physical attribute mentioned above, we classified all information based on four aspects: population characteristics, reported on-ice tests, outcome measures and main findings.

### 2.5. Population

For each study retained, we recorded the population's age (mean), sex, level of play and geographic location. For the mean age, the standard deviation was not considered for classification into the four age groups, which were categorized based on the Long-Term Athlete Development model (LTAD) (55): (1) under 12 years; (2) 12–15 years (youth); (3) 16–19 years (early expertise); and (4) 20 years and +(advanced expertise). In cases involving more than one age group, each subgroup was considered in the number of studies. Sex was classified as male and female. Three playing levels were categorized: youth hockey, competitive amateur (college, university and junior), and professional level. Studies including more than one playing level were classified for all levels under study. The variable “place” was classified according to geographic context: Europe, North America and other (Asia and Australia).

### 2.6. Types of associations

Types of associations were analyzed to specify the context in which studies were conducted and were classified based on selected studies' outcomes measures: physiological variables, talent identification, training effects (e.g., following interventions), validation, test parameters, off-ice testing, and other measures (e.g., biomechanical, impact of equipment/nutrition). The authors noted three categories of research designs: observational, (quasi) experimental and validation studies. Researchers gathered all information by formatting an Excel document to include these details. They then extracted the information by attributing a numeric code to each article to classify and analyze the distribution of each type of study.

## 3. Results

### 3.1. Selection of articles

Figure 1 illustrates the PRISMA procedure followed for article selection. A total of 1,504 articles were found through a search of three databases: PubMed (*n* = 394), SPORTDiscus (*nn* = 439) and Scopus (*n* = 671). The combined database search yielded 849 titles after removal of duplicates, and 18 studies were added manually (Figure 1). Analysis of the titles and abstracts of each article resulted in the identification of 148 studies for full text review. Among these, 23 studies were excluded for failure to meet quality assessment criteria. At completion of the qualitative analysis process, 107 studies met all the eligibility criteria and were included in the statistical analyses, resulting in 55 on-ice tests. Table 1 provides a summary of the articles in the literature: a total of 107 articles representing 55 on-ice protocols. As displayed, results indicate that tests were designed for assessing aerobic capacity (*n* = 7), skating acceleration and speed (*n* = 6), agility-changes of direction (*n* = 23) and repeated skate sprint ability (*n* = 19).

**Table 1:** Summary of articles reviewed.

			Tested attributes			
	Observed characteristics	Total articles	Aerobic	Acc-Speed	Agility	RSSA
	# Articles (*n*)	107	21	66	47	41
	# Tests (*n*)	55	7	6	23	19
Age	<12 years	3	0	2	2	1
	12–15 years	21	4	18	16	4
	16–19 years	39	9	24	15	20
	>20 years	59	12	34	23	24
Sex	Male	95	21	58	42	34
	Female	27	4	19	12	8
Playing level	Youth hockey	34	5	26	21	7
	Amateur	58	11	33	20	22
	Professional	22	7	13	10	11
Place	North America	57	11	36	24	24
	Europe	48	10	29	21	16
	Other	2	0	1	2	1
Design	Observational	69	13	45	34	28
	Experimental	22	4	13	10	9
	Validation	16	4	8	3	4
Aims-Outcomes	Physiological variables	38	5	23	15	19
	Talent identification	19	4	16	15	8
	Training effects	17	2	11	7	6
	Validation	16	4	8	3	4
	Test parameters	5	2	1	3	3
	Off-ice testing	4	3	0	0	1
	Others	9	1	7	4	0

Acc-Speed, acceleration and speed; Agility, agility and change of direction; RSSA, repeated skating sprints ability

### 3.2. Descriptive results

Table 1 examines a general overview of the included articles, with emphasis on each tested attribute and study parameters (i.e., age, sex, level of play, location, type of associations).

#### 3.2.1. Population characteristics

##### 3.2.1.1. Age

For the populations studied, the age groups most frequently tested in the scientific literature included players over 16 years old, divided into those over 20 years (*n* = 59) and those 16–19 years (*n* = 39). Players under 12 years and 12–15 years old were less tested (respectively *n* = 3; *n* = 21). Specific to the categories of athletic attributes, older cohorts (e.g., ≥16 years old) were most frequently assessed for on-ice acceleration-speed and ability to repeat skating sprints. Conversely, younger groups of athletes (e.g., <15 years old) were mainly tested on skating agility and on-ice acceleration-speed attributes.

##### 3.2.1.2. Sex and playing level

Regarding sex, males were by far most frequently tested (*n* = 95) compared to females (*n* = 27), for each tested attribute. Some differences were observed according to level of play, where results indicate that the literature focused more on amateurs (*n* = 58) than youth or professional level players (respectively *n* = 37 and *n* = 22). On-ice speed tests were mainly administered among amateur cohorts (*n* = 33), youth assessment focused more on speed and agility components (*n* = 26; *n* = 21), and professional athletes were tested in similar proportions on all physical attributes.

##### 3.2.1.3. Geographic location

Results show that geographic location was well distributed. More on-ice evaluations were conducted in North America (*n* = 57) compared to Europe (*n* = 49), and only two studies were carried out in other countries (Asia and Australia).

#### 3.2.2. Design

Most of the research done in on-ice hockey testing consisted of observational studies (*n* = 69), while experimental and validation protocols were less frequent (respectively *n* = 22 and *n* = 16). These results are similar when the focus shifts to each specific attribute, with a primary focus on on-ice acceleration and sprinting qualities over other attributes.

#### 3.2.3. Aims and outcomes

Results demonstrate that the main objective of research conducted in ice hockey on-ice assessment relates to physiological variables (*n* = 38). Below, talent identification, training effect and validation are subsequent outcomes showing similar proportions (*n*_talent_ = 19; *n*_training_ = 17; *n*_validation_ = 16). Then, test parameters, off-ice testing and other studies objectives were implemented in the same ratio (respectively *n* = 5; 4 and 9). On-ice acceleration and speed components along with agility were the two major athletic attributes assessed in the focus on on-ice testing in hockey research.

### 3.3. Summary of articles included in the systematic review

Table 2 presents results from all the retained articles that focused on on-ice testing in ice hockey. For each article, specifications in regard with population characteristics, reported test, outcome measures and main findings are presented.

**Table 2:** Basic characteristics of included articles focused on on-ice hockey testing.

Authors	Population characteristics (age, sex, level, place)	Reported test(s)	Outcomes measures	Main findings
(56)	12–15 years, male (M), recreational, North America (*n* = 24)	30.5 m sprint, 30.5 m backward, skating agility test with and without a puck	Estimate oxygen cost during on-ice testing.	Regression equation to estimate the oxygen cost for the 4 tests which the correlation coefficient is ranged from 0.91 to 0.93.
(57)	12–15 years, M, recreational, North America (*n* = 26)	Skating Multistage Aerobic Test (SMAT)	Validation of V˙O2max during the SMAT.	The use of the skating stride index in the regression equation improves the prediction of V˙O_2_max by reducing SEE by over 10%.
(58)	12–15 years, M, recreational, North America (*n* = 18)	30 m sprint, 30 m backward, skating agility test with and without a puck, skating anerobic power	Evolution of morphological, physiological, and on-ice performance.	All on-ice performance improved during the season. During the off-season, no improvement was observed and there was a deterioration of backward skating.
(59)	20 + years, M, professional, Europe, (*n* = 24)	Repeated skating sprint ability test, Skating Multistage Aerobic Test	Determine the effect of two rests period (2 min and 3 min) during a repeated sprint ability test.	The RSSA-3 causes players to skate faster, to reduce average heart rate (HR) and RPE, and to increase blood lactate comparing RSSA-2.
(60)	20 + years, M, amateur, North America (*n* = 30)	Short radius test turn, cone agility test, full speed	Relationship between off-ice testing and skating performance.	Forty yards sprint and balance ratio are correlated with full speed test (*r* = 0.51).
(61)	20 + years, M, amateur, Europe (*n* = 10)	30-15 intermittent ice test (30-15 IIT)	Examine the cardiorespiratory responses during 30-15 IIT	Lactate peak was higher after the on-ice version of the test compared to the running version. The HR peak and V˙O_2_peak were lower.
(62)	20 + years, female (F), amateur, North America (*n* = 20)	Repeated 10 m sprint, Reed repeated skate sprint test (RSS)	Relationship between on-ice performance, off-ice testing, and game performance.	Lower body fat percent and body mass enhance better performance in RSS while short length sprint is correlated with game performance (assists). Wingate variables are positively correlated with RSS.
(63)	16–19 years, M, recreational, North America (*n* = 40)	Full speed, acceleration 15.24 m, multiple repeated sprint time test	Reliability of the three on-ice testing.	The three tests showed a good reliability between days (ICC = ≥0.83).
(64)	20 + years, M, professional, North America (*n* = 33)	Sargeant anaerobic skate (SAS), modified repeated skate sprint test (RSS_m_)	Relationship between broad jump, vertical jump and two anaerobic tests.	For defensemen, the SAS is correlated with vertical jump while forward performance is correlated with broad jump. No correlation was found with RSS_m_.
(28)	16–19 years and 20 + years, F, amateur and professional, North America (*n* = 23)	6.1 m acceleration, 47.85 m sprint, full speed, cornering S turn, Reed repeated skate sprint test (RSS)	Comparing elite and non-elite on-ice performance.	Elite players have higher performance at RSS and full speed test than non-elite players. No differences were observed during the acceleration, 47.85 m sprint and cornering S turn.
(5)	U12y, M and F, recreational, North America (*n* = 54 F and 77 M)	6.1 m acceleration, 44.8 m sprint, cornering S turn	Comparing M and F on-ice performance.	M have better performance in the 44.8 m sprint and in the cornering S turn than F.
(65)	12–15 years, F, recreational, North America (*n* = 61)	6.1 m acceleration, 47.75 m sprint, cornering S turn, modified repeated skate sprint	Relationship between off-ice testing and on-ice performance.	Forty yards sprint is a strong predictor of 47.75 m sprint. Vertical jump is correlated with modified repeat skate.
(66)	20 + years, M, professional, Europe (*n* = 14)	Repeated skate ability 8 × 20 m (RSA)	Determine the effect of a high intensity shock microcycle.	High intensity shock microcycle improves the total time at RSA.
(67)	16–19 years, M, recreational, Europe (*n* = 17)	30-15 IIT	Validity and reliability of 30-15 IIT.	30-15 IIT is a reliable and a valid test. ICC ≥ 0.94 for all HR measurements.
(32)	20 years+, M and F, amateur, North America (*n* = 9 M and 10 F)	34 m sprint	Compare full body center of mass speed curve through the skate start to maximal speed between high caliber M and F.	M had better peak skating speed, greater hip abduction and knee flexion than F.
(68)	20 years+, M, recreational and amateur, North America (*n* = 18)	10 m acceleration, 20 m acceleration and 30 m sprint	Reliability of a wireless and portable system to measure biomechanics variables and compare acceleration and steady-speed.	Coefficient of multiple correlation was >0.65 for the reliability of the electromyography measurements of all muscles. Gastrocnemius were more activated during the acceleration phase while vastus lateralis and vastus medialis were more activated during the steady-speed phase. High calibers perform better than lower calibers.
(69)	16–19 years, M, recreational, Europe (*n* = 14)	10 m acceleration, sprint 30 m	Relationship between physical fitness and on-ice external load.	No significant correlation was found between on-ice test and the external load variables using a local positioning system.
(70)	16–19 years, F, amateur, North America	3 × 5 laps with 30 s rest in between	Relationship between V˙O_2_max and high intensity intermittent on-ice test.	V˙O_2_max was not correlated with the ability to recover from high intensity intermittent test.
(29)	20 + years, M, amateur, North America (*n* = 15)	15.25 m sprint, 35.05 m sprint	Validation of the polar team pro system.	Polar pro system underestimate speed for the 15.25 m and 35.05 m sprint by 23.1% and 37.5% respectively.
(71)	20 + years, M and F, amateur, North America (*n* = 15 M, *n* = 18 F)	148 m on-ice skating test	Comparing position performance during on-ice test.	No difference was observed between forwards and defensemen for the on-ice skating test. Body fat is correlated with the change of direction and total skating time.
(9)	16–19 years, M, amateur, Europe (*n* = 18)	Skating Multistage aerobic test (SMAT), 10 m acceleration, 35 m sprint	Determine the effect of combined plyometric and strength training program.	Adding plyometric to a strength training improves 10 m acceleration time but not 35 m sprint time. No difference was observed for the SMAT.
(72)	16–19 years, M, amateur, North America (*n* = 21)	SMAT, 7.65 m acceleration forward and backward, 30.48 m forward and backward sprint	Relationship between fitness, on-ice performance, and game performance.	Broad jump is associated with skating speed. Forward sprint is associated with expected goals.
(21, 73)	20 + y, M, amateur, North America (*n* = 18)	30 m sprint, 30 m backward, on-ice pro-agility	Relationship between on-ice and off-ice tests.	Standing long jump, vertical jump and Wingate relative peak power are correlated with on-ice 30 m sprint.
(74)	20 + y, M, amateur, North America (*n* = 12)	On-ice graded exercise 80 s-40 s	Comparing on-ice and off-ice graded exercise.	V˙O_2_max, blood lactate threshold and maximal HR are higher when the test in perform on the ice. No correlation was observed when comparing the V˙O_2_max between the off-ice and on-ice test.
(75)	16–19 years and 20 + years, M and F, amateur, North America (*n* = 10 M and 10 F)	On-ice graded exercise 80 s-40 s	Comparing V˙O_2_max and lactate threshold between sex.	V˙O_2_max is higher for M than F while F has a higher ventilatory threshold. Ventilatory threshold should not be used to predict lactate threshold for both sexes.
(76)	20 + years, M, amateur, North America (*n* = 16)	On-ice graded exercise 80 s-40 s	Examine the changes for maximal aerobic capacity and lactate threshold throughout a hockey season.	V˙O_2_max decrease from pre-to postseason.
(77)	12–15 years and 16–19 years, M and F, recreational, North America (*n* = 525 M and 71 F)	6 laps endurance test, 100 feet sprint, 100 feet backward, weave with and without a puck, transition agility with and without a puck	Relationship between on-ice performance and concussion.	Hockey Canada skills test are reliable test for hockey players. Players with and without concussion history have similar results in these on-ice tests.
(78)	16–19 years, M, amateur, North America (*n* = 18 M and 2 F)	35 m sprint	Determine the effect of specific off-ice training using SkateSim.	SkateSim improves more the 35 m sprint than regular plyometric training, even if both training improve the on-ice performance.
(79)	16–19 years, M, recreational, North America (*n* = 36)	35 m sprint, Cornering S turn	Relationship between off-ice and on-ice test.	Thirty-five meters sprint and cornering S turn are strongly correlated. Thirty meters off-ice sprint and three hops jumps are the best predictor for 35 m on-ice sprint.
(80)	20 + years, M, amateur, North America (*n* = 15)	8× tight turns	Comparing skate sharpening with three different hollow radii on on-ice performance.	No difference was observed between the blades sharpened with hollow radii ranging 9.53 mm to 22.23 on on-ice performance. Players with hollow radii of 3.18 mm had slower time.
(81)	20 + years, M, amateur, North America (*n* = 12)	10 m acceleration, 45 m sprint, 5× tight turns	Effect of a flared blade design on on-ice performance.	Players with the flared blade design were faster in all three tests compared to a standard blade.
(82)	20 + years, M, professional, North America (*n* = 162)	Skating Multistage aerobic test, 30-15 intermittent ice test	Comparing V˙O_2_max between generation, league level and position.	No difference for the V˙O_2_max between generation, league level and position. The study suggests that 55.9 ml/kg/min O_2_ is a requirement to play at professional level in North America.
(16)	20 + years, M and F, no level mentioned, North America (*n* = 10 M and 4 F)	5 m acceleration, 40 m sprint	Determine the reliability and validity of Kinexon local positioning system (LPS)	Kinexon LPS is reliable for the majority of LPS measures with ICC > 0.90. It showed a good validity when compared with robotic sprint device for peak speed, speed at 5 m and 0–5 acceleration.
(83)	20 + years, F, amateur, North America (*n* = 192)	6.1 m acceleration, 44 m sprint, cornering S turn, modified repeated skate sprint (MRSS)	Anthropometrics and off-ice tests to predict on-ice performance.	Forty yards sprint and sit-up are a good predictor for on-ice acceleration, sprint and MRSS. Higher mesomorphy, biacromial breadth, lower endomorphy and lower body mass index is correlated with MRSS and 44 m sprint.
(84)	20 + years, F, amateur, North America (*n* = 112)	6.1 m acceleration, Cornering S turn, modified repeated skate sprint (MRSS)	Comparing physical and performance characteristics between positions.	Forwards had better time than defensemen for the MRSS. No difference between forward and defensemen was observed for agility.
(85)	20 + years, M and F, professional, Europe (*n* = 10 M and 11 F)	6.1 m acceleration, 47.85 m sprint, full speed, cornering S turn	Relationship between physiological and on-ice performance.	For F, acceleration and 47.85 m sprint were correlated positively with body weight and negatively correlated with blood lactate accumulation and respiratory exchange ratio. For men only V˙O_2_peak was associated with 47.85 m sprint.
(86)	U12 and 12–15 years, M, recreational, Europe	20 m sprint, 30 m sprint, manœuvre skating	Determine technical training model to improve on-ice performance.	Greatest improvement was observed for the manoeuvre skating test and backward skating in the initial training period while basic training period and specific training period improved backward skating and 30 m sprint.
(87)	16–19 years, M, amateur, Europe (*n* = 19)	Skating Multistage Aerobic test, 5 m acceleration forward and backward, 10 m acceleration forward and backward, 30 m sprint forward and backward, full speed, Reed repeated skate sprint test	Relationship between vertical jump and on-ice skating tests.	Countermovement jump (CMJ) was correlated with backward skating. Squat jump showed a strong association with backward and full speed. Depth drop jump was correlated with forward sprint, backward sprint, and full speed.
(88)	12–15 years and 16–19 years, M, recreational, Europe (*n* = 10)	40 m sprint, Specific overall skating performance test	Developing a specific overall skating performance test.	SOPST show a high test-retest reliability with ICC = 0.7; CV = 5%. The test is well suitable to be used at different age (U20 < U17 < U15).
(89)	16–19 years, M, amateur, Europe (*n* = 15)	36 m sprint, NHIF agility test	Relationship between off-ice test and on-ice performance.	On-ice 36 m sprint was correlated with off-ice 36 m sprint and CMJ.
(90)	12–15 years, M, amateur, Europe (*n* = 18)	36 m sprint, 36 m backward, NHIF agility test	Relationship between off-season power changes and in-season skating changes.	Broad jump and 36 m running changes during off-season were correlated with 36 m on-ice sprint.
(91)	16–19 years, F, professional, Europe (*n* = 23)	Cornering S turn, cone agility, transition agility, modified repeated skate sprint	Relationship between off-ice tests and on-ice performance.	Single leg long jump was the best predictor for Cornering S turn, transition agility and MRSS.
(92)	16–19 years, M, amateur, Europe (*n* = 24)	Specific on-ice repeated sprint ability test	Reliability of on-ice repeated sprint ability and agility.	Sprint decrement (ICC = 0.78), Total time (ICC = 0.97), Best time (ICC = 0.98).
(93)	20 + years, M, amateur, North America (*n* = 20)	Repeated on-ice sprint test (ROIST)	Difference between positional in matter of fitness.	Forward had higher heart peak during the ROIST than defensemen.
(94)	12–15 years, M and F, recreational, North America (*n* = 20)	6.1 m acceleration, full speed, 44.8 m sprint	Determine the effect of BungeeSkate on on-ice speed and acceleration.	BungeeSkate training significantly increased 44.8 m sprint and full speed performance and slightly improved acceleration.
(95)	20 + years, M and F, amateur, North America (*n* = 15 M and 11 F)	6.1 m acceleration, 44.8 m sprint, full speed, cornering S turn, Reed repeated skate sprint	Relationship between off-ice tests and on-ice performance.	Forty yards sprint, vertical jump, 1.5 miles run, and percentage drop were correlated with MRSS and 44,8 m sprint.
(96)	16–19 years and 20 + years, M, amateur, Europe (=12)	30 m sprint	Describe lower limb activation during an on-ice sprint and examine his relationship with skating speed.	Uniarticular hip, knee and ankle extensors are activated during the propulsion phase whereas ankles dorsiflexors are activated during the recovery phase. No association were found between muscle activation and skating speed except for the negative correlation observed with the maximus gluteus.
(97)	20 + years, F, professional, Europe (*n* = 18)	11 m acceleration, 34 m sprint	Determine the effect of High-Intensity interval training on on-ice performance.	High-intensity training did not improve on-ice performance.
(98)	12–15 years, M, recreational, Europe (*n* = 33)	40 m with change of direction	Determine the effect of plyometric training on skating performance.	Plyometric training had a small effect on 40 m change of direction.
(99)	12–15 years, M, recreational, Europe (*n* = 33)	10 m acceleration	Determine the effect of plyometric training on skating performance.	Plyometric training did not improve on-ice 10 m acceleration test.
(100)	16–19 years, M, recreational, North America (*n* = 38)	34.5 m sprint, short radius turn, crossover turn	Relationship between off-ice and on-ice performance.	Forty yards sprint is the best predictor of 34.5 m sprint, short radius turn, and crossover turn.
(10)	16–19 years, M, amateur, North America (*n* = 41)	9 × 40 m sprint	Determine the effect of contrast training on on-ice repeated sprint ability.	Contrast training 6 h prior post-activation potentiation improved total time, mean sprint speed and 1st sprint speed in an RSA test.
(101)	20 + years, M, amateur, North America (*n* = 45)	Repeated shift test	Relationship between skating economy and fatigue during repeated shift test.	V˙O_2_ and energy expenditure are correlated with total decrement score.
(102)	20 + years, M, amateur, North America (*n* = 18)	7× shift test	Determine the effect of active and passive recovery on lactate concentration and on-ice performance.	No difference was observed for lactate concentration between active and passive recovery. Small difference was observed for the skating distance during the second set of the test.
(103)	16–19 years and 20 + years, M, not mentioned, Asia (*n* = 20)	T-test, 5× rink dash, 5 × 18 m shuttle, line drill	Determine the effect of complex training on skating abilities.	Complex training improved on-ice performance in the four on-ice tests.
(104)	16–19 years, M, junior, Europe (*n* = 21)	Repeated ice shuttle sprint (RISS)	Comparing on-ice and off-ice repeated sprint test.	Off-ice repeated sprint does not predict with enough precision on-ice repeated ice shuttle test.
(11)	12–15 years, M and F, recreational, North America (*n* = 86 M and 113 F)	6 m acceleration, 44 m sprint, Finnish Vierumaki agility test	Analyze discriminant validity of multi-dimensional talent identification testing protocol.	Boys had better on-ice performance than girls. Multi-dimensional talent identification testing protocol was discriminant for girls but not for boys.
(105)	12–15 years, M and F, recreational, North America (First part: *n* = 30 M, second part: *n* = 112 M and 31 F).	Skating Multistage Aerobic Test (SMAT)	Validity of SMAT.	SMAT showed a good reliability (*r* = 0.92, SEE = 0.56 stage). Low correlation was observed between 20 m shuttle run and the SMAT which means that SMAT is a reliable, valid and a specific test to predict V˙O_2_max. Players reach a higher V˙O_2_max during the SMAT.
(106)	20 + years, M, professional, North America (*n* = 36)	Yo-Yo IR1	Determine the effect of training status on on-ice performance and game-induced muscle damages.	Cardiovascular loading during the Yo-Yo is correlated with total high-intensity, very fast speed skating and number of high intensity bouts during a game.
(107)	20 + years, M, amateur, North America (*n* = 43)	Repeated shift test	Association between ventilatory threshold and repeated shift test.	Ventilatory threshold is a stronger predictor than V˙O_2_peak for the repeated shift test. Ventilatory threshold is strongly correlated with gate 2 during the test.
(108)	20 + years, M, level not mentioned, North America (*n* = 11)	30 m sprint forward and backward, weave agility, transition agility, reaction drill	Effect of caffeine on on-ice performance.	No significant effect was observed for low dose of caffeine on on-ice performance except for the transition agility test.
(18)	12–15 years, M and F, recreational, North America (*n* = 86 M and 113 F)	6 m acceleration forward and backward, 44 m sprint forward and backward, Finnish Vierumaki agility test	Associations between off-ice testing and on-ice performance and team selection.	Height or body mass had no significant effect on on-ice performance.
(13)	12–15 years, M, recreational, North America (*n* = 59)	6 m acceleration, 44 m sprint, transition skating agility (IIHF)	Relationship between on-ice testing protocol and game demands.	Exertion duration of the protocol (1 min) is like a game shift. HR during on-ice testing is comparable with game context.
(109)	20 + years, M, professional, Europe (*n* = 11)	25 m sprint	Determine the effect of resisted sprint on 25 m on-ice sprint.	Heavy resisted sprints are sufficient to induce an acute potentiation and improves 25 m sprint.
(110)	16–19 years, M, amateur, North America (*n* = 24)	33 m sprint, 6 × 9 stops, line drill	Determine the effect of high intensity training using Wingate anaerobic test on on-ice performance.	High intensity group improved the 33 m sprint and the line drill compared to control group.
(111)	20 + years, M, recreational, Europe (*n* = 20)	35 m sprint, cornering S turn.	Relationship between on-ice performance and game performance (plus and minus).	Plus and minus were not associated with on-ice test performance.
(49)	20 + years, M recreational to professional, North America (*n* = 24)	Pro-agility test	Reliability and validity of pro-agility test.	Reliability of pro-agility (ICC = 0.817).
(112)	20 + years, M, amateur, North America (*n* = 15)	3 × 4 min laps	Comparing skating economy and V˙O_2_ between on-ice and skating treadmill.	Stride rate and HR were higher on the skating treadmill than on the ice. V˙O_2_ was similar.
(50)	12–15 years, M, recreational, Europe (*n* = 14)	6.1 m acceleration, 35 m sprint, cornering S turn, test with break, weave agility, reactive agility test	Determine the effect of on-ice and off-ice agility training on skating performance.	On-ice and off-ice agility training are both beneficial to improve on-ice agility, but on-ice training has better improvement on weave and reactive agility test.
(113)	12–15 years, M, recreational, Europe (*n* = 13)	Transition agility with and without a puck, 4 m acceleration with and without a puck (forward and backward) and 30 m sprint with and without a puck (forward and backward).	Compare the effect of change of direction training program, speed exercises and partial skating task on skating performance.	Change of direction improved transition agility with puck and 4 m acceleration while partial skating task improved 30 m sprint and transition without a puck.
(114)	12–15 years, M, recreational, Europe (*n* = 40)	36 m sprint forward and backward, 6 × 9 m turns, 6 × 54 m	Determine the effect of powerskating on on-ice testing	Powerskating improved all on-ice tests compared to control group.
(115)	16–19 years, M, amateur, Europe (*n* = 20)	40 m with change of direction	Determine the effect of time and chronotype on speed abilities.	Speed abilities is higher in the afternoon than in the morning. Chronotype does not affect speed abilities.
(116)	20 + years, F, amateur and professional, Europe (*n* = 10)	20 m sprint with 75% body mass, 25 m–30 m sprint with 50% body mass, 40 m sprint with 3 kg	Compare force and velocity relationship during multiple loaded skating sprint and a single unloaded skating sprint.	High to very high correlations were observed for all mechanicals characteristics (F_0_, V_0_, P_max_, SFV and V˙O_pt_) between multiple loaded skating sprint and unloaded skating sprint.
(38, 116)	16–19 years, F, recreational, Europe (*n* = 11)	5 m acceleration, 40 m sprint	Reliability of radar-derived profiling from skating sprint acceleration.	All mechanical variables had ICC≥0.75; CV≤10% for the test-retest reliability except for the S_fv_ and S_fvrel_.
(117)	20 + years, F, professional, Europe (*n* = 17)	5 m acceleration, 40 m sprint	Associations between on-ice and off-ice mechanical capacities derived from force and velocity relationship.	RF_max_, F_0rel_ and P_maxrel_ are the main mechanical variables associated with the 5 m acceleration and 40 m sprint. Forty meters sprint was correlated with 30 m running sprint and squat jump, but not the 5 m acceleration.
(39)	16–19 years and 20 + years, M, amateur, Europe (*n* = 45)	15.24 m sprint, full speed, repeated shift test	Relationship between off-ice anaerobic power and on-ice tests.	Vertical jump and Wingate anaerobic test are correlated with 15.24 m sprint and full speed. Off-ice aerobic test does not predict repeated shift performance except for the fastest course time.
(118)	16–19 years and 20 + years, M, amateur, Europe (*n* = 45)	15.24 m sprint, full speed, repeated shift test	Comparing DI players, elite junior and DIII players for off-ice and on-ice performance.	Division 1 players had greater full speed time and fastest repeated shift test course time.
(119)	16–19 years and 20 + years, M, amateur, Europe (*n* = 45)	15.24 m sprint, full speed, repeated shift test	Relationship between V˙O_2peak_ and repeated shift test.	V˙O_2_peak was correlated with gate 2 speed decrement (%). The final stage of graded skating treadmill test was correlated with gate 2 and total course speed decrement (%).
(120)	12–15 years and 16–19 years, M and F, recreational and amateur, North America (*n* = 291 M and 115 F)	Faught aerobic skating test	Validity of Faught aerobic skating test.	Regression model including height, body mass, age and fast length to predict the V˙O_2max_ (*R*^2^ajusted = 0.387; SEE = 7.25 ml.kg^−1^.min^−1^, *p* < 0.0001).
(121)	20 + years, M, amateur, North America (*n* = 24)	Dot to dot sprint, lap test, shortlightning test	Relationship between on-ice, off-ice and game performance.	No correlation was found between on-ice test performance and game performance.
(122)	20 + years, M, amateur, North America (*n* = 21)	Reed repeated skate sprint test	Relationship between body composition, leg strength, anaerobic power and repeated skate sprint test.	First length skate average and total length skate average were moderately correlated with % of body fat. Wingate % fatigue was correlated with the fastest first length time while the peak power per kg was correlated with the first length average time.
(123)	U12y, M, recreational, North America (*n* = 14)	Repeat ice skate test (RIST)	Reliability and concurrent validity of the repeat ice skate test (RIST)	Reliability measuring average peak power in watt and watt per kg was *r* = 0.99 and 0.98 respectively. The test is valid when compared to vertical jump (*r* = 0,86), Margaria–Kalamen stair test (*r* = 0.66) and Wingate anaerobic test (*r* = 0.86).
(124)	20 + years, M, professional, Europe (*n* = 17)	Yo-Yo IR1,10 m acceleration, 30 m sprint, repeated sprint ability (3 × 30 m)	Association of on-ice test with game performance.	No association was found between on-ice tests and the work rate during a game.
(125)	16–19 years and 20 + years, M, amateur, Europe (*n* = 21)	30 m sprint forward and backward, 6 × 9 m turns and stops, endurance 6 × 30 m	Relationship between aerobic and anaerobic off-ice test and on-ice performance	Wingate relative peak power and V˙O_2_max are correlated with 6 × 9 turns and endurance 6 × 30 m.
(126)	12–15 years and 16–19 years, M, recreational, Europe (*n* = 60)	30 m sprint forward and backward, 6 × 9 m turns and stops, endurance 6 × 30 m	Physiological, fitness profile and on-ice performance to predict team selection.	No difference was observed for on-ice performance between players who have been selected on the team and the ones who were not.
(127)	20 + y, M, professional, Europe (*n* = 24)	30 m sprint forward and backward, 6 × 9 m turns and stops, endurance 6 × 30 m	Differences between physical fitness and skating performance when relegated in a lower league.	No significant differences were observed for 30 m forward, backward sprint, and 6 × 9 turns and stops. Players who were relegated in a lower league had worst results in the endurance 6 × 30 m.
(128)	20 + years, M, professional, Europe (*n* = 42)	30 m sprint forward and backward, 6 × 9 m turns and stops, endurance 6 × 30 m	Relationship between physiological, physical, and on-ice performance on team selection.	Players selected on the team perform better in 30 m forward sprint, 6 × 9 turns, 6 × 9 m stops and endurance 6 × 30 m.
(129)	16–19 years, M, amateur, Europe (*n* = 20)	Skating Multistage Aerobic Test (SMAT)	Effect of short and long interval training on on-ice performance.	Short interval training induced better performance than long interval training at the SMAT.
(130)	Not mentioned, M, amateur, North America (*n* = 40)	90 feet forward and backward sprint, full speed	Associations between off-ice tests and on-ice speed.	Vertical jump was correlated with 90 feet forward sprint.
(131)	20 + years, M, professional, Europe (*n* = 18)	Ice hockey-specific complex [Shots on goal, 10 m sprint and 30 m sprint (forward and backward), weave agility, shot on goal]	Examine the validity of ice hockey-specific complex by comparing with an off-ice test.	Weave agility with the puck is the most valid on-ice test for the match performance score (*R*^2 ^= 0.39).
(132)	20 + years, M, amateur and professional, Australia (*n* = 13)	5 m acceleration, 10 m acceleration, 20 m sprint, on-ice pro-agility	Relationship between joint angle, countermovement jump (CMJ) and on-ice performance.	CMJ relative peak force combine to 25° hip abduction explained 46% of 10 m acceleration time while CMJ peak eccentric velocity combine to 50° hip adduction explained 86% of pro-agility test.
(133)	20 + years, M and F, amateur, North America (*n* = 9 M and 10 F)	15 m acceleration	Evaluate the body movement kinematics during a skating start between M and F.	M had wider steps and greater hip abduction than Fs during the 15 m acceleration. M had a better peak skating speed than F.
(134)	16–19 years, M, amateur, Europe (*n* = 91)	5 × 54 m	Comparing anaerobic capacity between U16-U18, U20 and senior.	Difference between time during the 5 × 54 m test was observed (Senior < U20 < U18 < U16). Wingate relative maximum power was correlated with 5 × 54 m times for the Senior and U20 group.
(135)	16–19 years, M, amateur, Europe (*n* = 32)	On-ice Illinois with and without a puck	Relationship with off-ice, anthropometric and on-ice agility.	The off-ice agility test, and the Illinois with and without a puck were positively correlated. Skeletal robustness in the lower limb seems to decrease the performance in on-ice agility test with a puck.
(136)	16–19 years, M, recreational, North America (*n* = 22)	Repeat ice skate test (RIST), speed crossover (3 laps), cadence crossovers (3–5 laps)	Determine the effect of flywheel training on on-ice performance.	Flywheel did not improve on-ice testing.
(52)	20 + years, M, professional, Europe (*n* = 19)	Skating Multistage Aerobic Test, Repeated skate sprint ability (RSSA)	Determine the effect of recovery time (two minutes vs three minutes on repeated skate sprint ability.	Three minutes recovery is more beneficial for increasing skate speed during RSSA. No effect was found on speed decrement (%) and HR peak..
(137)	20 + years, M, professional, Europe (*n* = 24)	Reed repeat skate sprint (RRSS)	Relationship between aerobic capacity and fatigue during repeated skate sprint.	Test-retest (*r* = 0.78). V˙O_2_max and fatigue index for total length skate were correlated.
(138)	20 + years, M, amateur, North America (*n* = 28)	On-ice game simulation test (6 × 5 s active and 5 s for a total of 60 s)	Relationship between maximal aerobic power and recovery during the on-ice game simulation.	There is no relationship between maximal aerobic power and recovery during an on-ice simulated game test.
(139)	16–19 years and 20 + years, M, amateur, Europe (*n* = 12)	30 m sprint	Validity and reliability of split time method using high speed video camera to measure force and velocity profile.	The reliability and validity of the simple cost method is moderate. The use of time shift increased the reliability to excellent (ICC > 0.918),
(140)	20 + years, M, recreational and amateur, North America (*n* = 22)	30 m sprint	Determine biomechanical changes in skating performance using an accelerometer.	High caliber players had lower contact skating time, lower total sprint time and higher strides propulsions than lower caliber players.
(30)	20 + years, M and F, amateur, North America (*n* = 24 M and 22 F)	15 m sprint	Compare off-ice fitness tests and off-ice resisted sprint to predict 15 m on-ice sprint.	Off-ice resisted sprint with 15 and 30 kg are the best predictor among all off-ice tests for the on-ice 15 m sprint (*r* ≥ 0.70)
(12)	20 + years, M, professional, Europe (*n* = 298)	Yo-yo IR1, 10.5 m acceleration, 33.15 m sprint, on-ice pro-agility	Comparing fitness profile of elite and sub elite players.	Elite players outperformed sub elite in every on-ice tests.
(141)	16–19 years, M, amateur, Europe (*n* = 30)	3 × 33.15 m sprint	Examine muscle metabolism and fatigue during a simulated match-play.	The three sprints time increased after the third period.
(142)	16–19 years and 20 + years, M, amateur and professional, Europe (*n* = 169)	Yo-Yo IR1, 10 m acceleration, 30 m sprint, on-ice pro-agility	Comparing fitness profiles and body composition between U20 and highest national level.	Elite Finnish players had higher performance in all on-ice testing than Danish elite and U20. U20 Finnish had similar results than Danish elite except for the 10 m acceleration where U20 Finnish players had better performance.
(143)	12–15 years and 16–19 years, M, recreational and amateur, Europe (*n* = 13)	6 m acceleration, 30 m sprint, specific overall skating performance test (SOSPT)	Comparing fitness profiles between different age (U15-U17–U20) and relationship between on-ice test and SOSPT.	Difference was observed for all on-ice performance except for 6 m acceleration between each group where the older group performed better. SOSPT was correlated with 30 m sprint for all groups and the 6 m acceleration for the U20 group.
(144)	16–19 years, M, recreational, Europe (*n* = 12)	15 m sprint, cornering S turn	Relationship between physical variables, on-ice testing and game performance.	Fifteen meters sprint was not correlated with any game performance. Cornering S turn for high level is correlated with defensive contribution.
(145)	20 + years, F, amateur, North America (*n* = 17)	7 × 15 m sprint (ROIST), repeated 5 m and 10 m acceleration	Development and reliability of ROIST.	No significant difference between the four trials was observed for the fastest sprint time, the mean time and total time of the seven sprints (ICC > 0.75). Forwards perform better than defensemen.

M, male; F, female; 30-15 IIT, 30-15 intermittent ice test; SMAT, skating multistage aerobic test; HR, heart rate.

#### 3.3.1. Descriptive results with physical attribute focus

Table 3 examines a specific overview of the most used on-ice protocols in the scientific literature for each physical attribute tested. Results present tests that appears three times or more in the literature.

**Table 3:** Classification of articles by tested attribute with emphasis on the most used on-ice protocols.

Test (*n*)		Age				Sex		Level			Place	
	# articles	<12 years	12–15 years	16–19 years	>20 years	M	F	Youth	Am.	Pro	N-A	Eur.
Aerobic (7)	21	0	4	9	12	21	4	5	11	7	11	10
SMAT	9	0	2	4	3	9	1	2	4	3	4	5
Yo-Yo IR 1	4	0	0	1	4	4	0	0	1	4	1	3
30–15 IIT	3	0	0	1	2	3	0	1	1	1	1	2
Graded exercise	3	0	0	1	3	3	1	0	3	0	3	0
Less than 3 (3)	3	0	2	2	1	3	2	2	2	0	3	0
Speed (6)	66	2	18	24	34	58	19	26	33	13	36	29
Forward sprint	55	2	17	19	26	47	17	23	26	12	28	27
Forward accel.	45	2	10	14	26	37	17	17	24	13	23	22
Backward sprint	18	1	9	5	6	18	2	8	6	3	9	9
Full speed	11	0	1	6	7	10	4	2	8	2	9	2
Backward accel.	5	0	2	2	1	5	1	2	2	1	3	2
Less than 3 (3)	1	0	0	0	1	1	0	0	1	0	1	0
Agility (23)	47	2	16	15	23	42	12	21	20	10	24	21
Cornering S	12	1	2	4	6	7	8	6	4	3	7	5
6 × 9 stops	5	0	1	3	3	5	0	1	2	2	1	4
6 × 9 turns	5	0	2	2	3	5	0	2	1	2	0	5
Pro-agility	5	0	0	1	5	5	0	1	4	4	2	3
Transition	5	0	3	2	1	4	2	3	0	1	4	1
Weave	4	0	2	1	2	4	1	2	0	1	2	2
Less than 3 (17)	24	1	11	8	8	23	4	12	11	1	12	11
RSSA (19)	41	1	4	20	24	34	8	7	22	11	24	16
Reed	6	0	0	3	4	4	3	0	4	2	4	2
RST	5	0	0	4	4	5	0	0	5	0	5	0
MRSS	5	0	1	1	3	1	4	1	2	2	4	1
Endurance	4	0	1	2	3	4	0	1	1	2	0	4
MRST	3	1	0	2	2	3	0	1	0	2	1	2
Line drill	3	1	0	2	2	3	0	2	0	2	2	0
Less than 3 (13)	17	1	2	7	9	16	1	4	7	4	8	7
Total (55)	107	3	21	39	59	95	27	34	58	22	57	49

M, male; F, female; Am, amateur; Pro, professional; N-A, North America; Eur, Europe; SMAT, skating multistage aerobic test; 30-15 IIT, 30-15 intermittent ice test; RSSA, repeated skating sprint ability; RST, repeated shift test; MRSS, modified repeated skate sprint; MRST, multiple repeated skate test.

#### 3.3.2. Aerobic capacity

A total of 21 articles were found including tests of on-ice aerobic capacity in ice hockey players. The authors listed seven different tests where the majority of studies involved ice hockey players who were 20 years and older (*n* = 12), were male (*n* = 21) and played at the amateur level (*n* = 11). No studies were found on the on-ice aerobic capacity of ice hockey players under 12 years old. Additionally, four studies assessed on-ice aerobic capacity in female ice hockey players. Two of these were designed to test validation, while the others discussed the differences in the physiological parameters of males and females during graded exercise (75). Skating Multistage Aerobic Test (SMAT) is the on-ice aerobic protocol that appears most frequently in articles in the literature, most often regarding the age ranges of 16–19 years (*n* = 4) and 20 years+ (*n* = 3). Athletes from the amateur and professional levels were most often tested for aerobic capacity. These studies focused almost exclusively on male players (*n* = 9).

#### 3.3.3. Acceleration-speed components

There are 66 articles in the present review, corresponding to six tests to assess on-ice acceleration and speed components in ice hockey. Forward acceleration or sprint are the most commonly used protocols (respectively *n* = 55; *n* = 45), followed by backward acceleration, backward sprint and full speed. As for skating distances, the most frequently applied protocols are the 6.1 m forward skating sprint test for on-ice acceleration and the 30 m for on-ice forward speed. All these are applied progressively for age cohort groups, with a prevalence among older athletes (*n* = 34 for 20-year-old group; *n* = 18 for 12–15 year-old group and *n* = 2 for ≤12 year-old group). From a level of play perspective, more tests were conducted at the competitive amateur level (*n* = 33) than at the youth (*n* = 26) or professional (*n* = 13) levels. This athletic attribute was more often assessed in North America than in Europe (*n*_America_ = 36; *n*_Europe_ = 29).

#### 3.3.4. Agility and change of direction abilities

On-ice agility and change of direction tests appeared 47 times in scientific publications. A high variability is observed in the selection of this category of tests, since 23 tests were found to evaluate this athletic quality, 17 of which appeared less than three times. Furthermore, there is a considerable gap in the literature regarding validation of agility tests, since only one test, the specific overall skating performance test (SOSPT), was validated over the timeframe of this review. The use of agility testing across the different studies was mostly targeted to male athletes (*n* = 42) aged 20 years and older (*n* = 23) playing at a competitive amateur level (*n* = 20). Only two studies were found regarding the assessment of agility in young ice hockey players under 12 years old. This on-ice attribute is assessed as much in North America as in Europe (*n*_America_ = 24; *n*_Europe_ = 22), while Asia has one study. On an individual basis analysis, the Cornering S turn is the most frequently used on-ice test (*n* = 12) and implemented in both sexes. This protocol is well documented in the literature across the age ranges of 12–15, 16–19 and 20+ years old (*n* = 2; 4; 6) and is similarly represented in all levels of play (*n*_youth_ = 6; *n*_amateur_ = 4; *n*_pro_ = 3).

#### 3.3.5. Ability to repeat skating sprints (RSSA)

Ability to repeat on-ice skating accelerations, sprints or intense effort tests are well documented in ice hockey assessment, with 41 articles corresponding to 19 different on-ice protocols. A substantial and similar variability in agility is also observed in the selection of this category of tests, since 13 of the 19 tests occurred less than three times. In the various studies, tests of skating sprint repetition ability involved mainly male athletes (*n* = 34) in an older population (i.e., 16–19 and over 20 years old; cumulated *n* = 44) compared to younger cohorts (under 12–15 and under 12 years old; cumulated *n* = 5) playing at a competitive amateur level (*n* = 22). From an individual perspective, the Reed test appears to be the most conducted test (*n* = 6), while others (i.e., repeated shift test, modified repeated sprint skating, endurance test, multiple repeated skate test, line drill) follow below (from *n* = 5 to *n* = 3).

### 3.4. Summary of on-ice protocols trends

Table 4 highlights observations and practical applications of usage trends for each physical quality assessed. SMAT is the most conducted test for on-ice aerobic capacity assessment and is both valid (*r* = 0.97) and reliable (*r* = 0.92) (105). Acceleration and speed variables are mainly assessed with 6.1 m forward skating and 30 m skating sprints with a high reliability (ICC ≥ 0.83, TE ≤ 0.5%) (63). The cornering S test is the most common protocol for evaluating on-ice agility and change of direction. Nevertheless, although there is no consensus on ability to repeat sprints, trends seem to establish that the Reed repeat sprint skate test and the repeated shift test are practical options for assessing this athletic component.

**Table 4:** Observation trends, benefits and constraints of the most used on-ice protocols.

	Test	General observations	Benefits and disadvantages
Aerobic capacity	SMAT is the most frequently used test throughout the scientific publications (*n* = 9), valid and reliable test (*r* = 0.92; SEE = 0.56 stages).	Ice hockey players reach higher V˙O_2_max and cardiorespiratory responses during on-ice aerobic test when compared to similar off-ice test. Males have higher V˙O_2_max values and lower ventilatory threshold than females. Elite players perform better at SMAT test than sub elite. Shorts intervals training induced more improvements during the SMAT than longer intervals. No significant correlation was found between speed testing and game performance, but it correlates with game intensity.	Pros: provides physiological measures (e.g., V˙O_2_max extrapolation, max HR, recovery HR) more precise than with skating treadmill or shuttle run test; test reproduces specific movements (on-ice skating pattern, stop and go); logistically easy to perform and low cost. Cons: doesn't replicate match requirements (accelerations and repeated accelerations interspersed with gliding recoveries).
Acceleration & speed	Forward acceleration (*n* = 48) and sprint (*n* = 42) are the tests most reported. Due to an on-ice speed test distance variability, the 6.1 m acceleration (*r* = 0.8) and 30 m sprint (*r* = 0.98) are the ones we recommend.	Off-ice sprints, jump test and Wingate relative peak power are strong predictors of on-ice sprint performance. Males are faster than females and elite players perform better in sprint and acceleration test than sub elite. Plyometric training and resisted sprints on and off the ice improve skating performance. No significant correlation was found between on-ice speed testing and game performance.	Pros: provides acceleration, flying acceleration and maximal velocity measures; and is logistically easy and quick to perform; analysis of on-ice power-force-velocity profile Cons: doesn't replicate most in-game situations aside from a hard forecheck or backcheck; starting from a dead start doesn't happen often during a typical shift; doesn't give the information about maintaining these high-speed levels under fatigue; cost of the equipment required to achieve valid and reliable measures can be quite restrictive (timing gates or radar).
Change of direction & agility	Cornering-S turn is the agility test usually used for research. Cornering S turn has a high reliability (*r* = 0.95).	Few studies were able to correlate off-ice testing with agility test. Single leg long jump, 36.5 m sprints and anthropometrics measures are the physiological variables predicting on-ice agility. Males are more agile than females while elite players were better than sub elite. Off-ice and on-ice agility training seems to improve on-ice agility test. Two studies were able to correlate agility tests with game performance (match performance score and defensive contribution).	Pros: easy and quick to perform; involves ice hockey movement patterns (acceleration, crossovers on both sides, deceleration); specific read and react assessment for agility test Cons: doesn't consider other skating patterns (braking, sharp turns, pivots, backward); path is already known by the players (familiarization), improvements and transfers to game performance might be less relevant comparing to agility (unpredictability context).
Repeated skating sprint ability	No consensus between Reed repeated skating sprint test and repeated shift test while females mostly evaluated with the modified repeated skating sprint test.	Repeated sprint test was often correlated with anthropometrics, Wingate variables, jump test and V˙O_2_max. Elite players perform better than sub elite. A three-minute recovery instead of two minutes allows the ice hockey players to skate faster during the repeated skate sprint ability. No game performance was correlated with the capacity to repeat skating sprints.	Pros: provides information about acceleration-speed and the ability to repeat these attributes under fatigue; reproduces in-game requirements (short-duration efforts interspersed with brief recovery, accelerations, braking at high velocities) Cons: protocol duration for one player might be time consuming; skating pattern selection (forward only vs combination), work/rest ratio, type and duration of rest might affect exhaustion reaching and results; logistics might be difficult to achieve valid and reliable measures (multiple timing gates, number of evaluators to assess a group of athletes).

## 4. Discussion

The general aim of this systematic review was to describe the extent to which evaluation protocols for different populations of ice hockey players have been used over the last twenty years. To this end, we identified four key categories of attributes related to ice hockey performance: aerobic capacity, speed, agility and ability to repeat intense efforts or sprints. Despite researchers' efforts so far to document the usefulness of assessing hockey players (22), this review provides a complete overview of the work carried out in the specific fields of on-ice testing in ice hockey. Because on-ice tests are specifically linked to the actions of hockey players, we believe that stakeholders (researchers, strength coaches, coaching staff, etc.) can benefit from such an inventory by relying on tests adapted to the populations of athletes with whom they work. Considering the evolution of this sport and the physical characteristics of top-level hockey players (45, 82, 146), we limited our search to work published over the last two decades. We also excluded test protocols designed to measure technical or tactical skills, since this category refers mainly to young developing athletes. In this respect, hockey federations in most countries have already developed a list of tests based on their strategic orientations in terms of sports development (54, 147).

Regarding the populations studied and their attributes, it is interesting to note that the inventory of on-ice tests offers observations consistent with the attributes observed, as illustrated in Table 1. First, the on-ice agility and change of direction component predominates in the studies conducted with the youngest populations (e.g., 12–15 years old). This result is logical, given this attribute is a key element for young players aiming to further develop their hockey expertise; it is, moreover, consistent with most models of sports development (148). Our results suggest that young athletes under 12 years of age are relatively rarely evaluated, which is logically linked to the long-term athlete developmental stages. Such is not the case for their counterparts aged 16 and over, where evaluation becomes predominant in the progressive development of ice hockey-specific expertise (149). In more advanced populations (e.g., age group, level of play), we observe the importance of measuring the ability to repeat sprints, which is in line with the relevance of this on-ice performance indicator at the highest levels of competition (50, 92). Nevertheless, the most recent studies increasingly emphasize anaerobic (or hybrid) processes and their potential impact on performance in a game or competition context (145, 150, 151). In line with such results, the anaerobic component remains an important part of identifying potential NHL players, as it was demonstrated by Heller and colleagues (152) who tested elite Czech players. Our results for the acceleration and speed component, with a major utilization of 6.1 m and 30 m skating distances, are consistent with those of a previous systematic review (41).

As for test reliability and validity, results reveal that most of the protocols move in two opposite directions. First, the inventory proposed by our review suggests that most of the tests have very satisfactory levels of reliability and validity, at least with the populations studied using these protocols. Examples include the SMAT, 30-15 IIT, Yo-Yo IR, forward skating acceleration and sprint, cornering-S turn agility test, or even some ice hockey-specific repeated sprint tests such as the 7 × 15 m or the SOSPT. Another considerable part of these tests, however, has not been validated or replicated through an objective scientific process. This is mainly the case for tests targeting agility or ability to repeat intense skating efforts. Given their relevance, particularly for assessing the development of young talent, it is vital to identify and develop specific standardized, reliable and valid on-ice protocols to maintain qualitative analysis and optimal procedures to track progress and enable comparisons. Despite the lack of scientific insight, there is a definite advantage to assess in a sport-specific context that resembles a real game as closely as possible if the test is designed consistent with sport requirements (11, 104). As shown in Table 3, it exists a variability depending on the attribute evaluated. Aerobic and acceleration-speed capacities are assessed with a small range of tests (respectively *n* = 7 and 6), with a majority of them that have been validated and used in most research, in a logical manner. Conversely, a large variability has been highlighted in CoD-agility and ability to repeat skating sprints attributes, with a wide range of tests (respectively *n* = 19 and 23) developed to assess theses capacities in ice hockey athletes. Assess capacities as CoD, agility and RSSA in a specific ecological perspective on the ice has some issues in contrast of aerobic or speed qualities, which seems to be easier to standardize.

Most of the tests considered in this systematic review do not include the puck in the assessment, as described in Table 2. We think that it is logical mainly for practical reasons because puck loss during on-ice testing would lead to the athlete being required to repeat the test, which would increase the total evaluation time. Pucks could also damage specific measurement systems such as photoelectric cells. Therefore, it would be necessary to implement these on-ice tests both with and without pucks, to assess potential puck control differences and provide better support for each athlete. However, the use of the puck in on-ice acceleration, speed, CoD and agility tests could reveal an interesting detail about the offensive aspect of players. For example, a player who can reach a high percentage of his maximum on-ice skating speed while controlling the puck, as compared to a team-mate who degrades his skating speed in same conditions, is also an essential factor to consider for training and performance purposes.

This review also reveals that female players has so far received very little attention in the field of on-ice testing compared to their male counterparts. Considering the different physical capacities and the different role they can play in the selection process for female athletes, a more specific focus on the development of tests specific to female hockey players could offer some interesting advancements (11). In other words, it may not be optimal to consider the skills of female hockey players similarly to those of males given the game is structured differently, which could lend greater importance to other aspects.

Regarding level of play, our results indicate that most studies focus on the amateur sphere, with less attention paid to professional and youth levels. Data sensitivity at elite levels limits the possibility of evaluating athletes for research purpose, information is confidential and restricted to staff members who cannot communicate results because of privacy data protection. At youth levels, however, a plausible explanation could be that the development of technical on-ice skills takes priority over evaluation when an athlete lacks the necessary technical prerequisites, making evaluation needless. Our analysis reveals that most studies of on-ice performance evaluation are descriptive. This is understandable considering it's easier to observe trends, relationships and correlations than to conduct more in-depth studies within structures, clubs and federations (e.g., longitudinal follow-up, effect of specific training on on-ice performance). Moreover, on-ice evaluation batteries are intended for formative or selective purposes.

### 4.1. Practical applications

The inventory proposed by this review helps identify the avenues to explore for a more enlightened view of the (evolving) status of ice hockey players worldwide. For researchers, it is a relevant guide that allows studies to be replicated based on the characteristics or variables being studied. An inventory of on-ice tests also gives researchers an indication of the populations under study. The tool enables teams' coaches to make informed choices that will facilitate analysis and interpretation of the standards achieved by the populations under study. Coaches can also use on-ice testing by integrating it as a drill during practices to develop each attribute in a high pace manner. For example, agility test ended by a shot can be used at the start of practice to develop skills and agility to mimic game specific situations. Another option might be using RSSA test at the end of the practice and include several shots during each recovery time to develop the ability to perform and maintain precision shots despite fatigue and enjoying more the conditioning aspect of the drill. For strength and conditioning coaches, the assessment of all four areas of physical attributes is relevant for designing adapted training programs and verifying the outcomes of specific ice hockey conditioning training. It could also help them to better identify and select the off-ice tests which are closest to the on-ice tests most frequently used in the literature, with the aim of providing assessments that are more tailored to athletes' needs.

### 4.2. Limitations and perspectives for future research

Despite the insights offered in this review, its limitations suggest avenues for future research. A first limitation is the identification of studies considering players' on-ice fitness-performance. Here we considered only those published in academic journals. Some interesting approaches might reside in other types of publication, such as unpublished theses, hockey federations' technical manuals or reports, and unpublished work. As an example, Hockey Canada has designed on-ice protocols and standards with a battery of tests, some based on the scientific literature and others that were empirically developed (54). Further research should also consider the on-ice tests designed and used mainly by federations and ice hockey clubs to analyze in depth the benefits of such assessments. However, the purpose of this study was to identify tests that were used and replicated in different research contexts. Additionally, we focused on a date range of the last twenty years of research (i.e., 2000–2002), a limitation in that we may have missed scientific articles published before that time, affecting proportions and results. Another limitation relates to emerging testing approaches that require advanced technologies such as global and local positioning systems (e.g., GPS, LPS), inertial movement units and object tracking methods. Since the reliability and validity of such technologies is well supported (16, 153), these promising approaches are now well established in soccer, while they have become more popular in the field of ice hockey research in recent years (36, 37, 154). From this perspective, we believe that such new and precise assessment methods will provide additional opportunities for researchers and professionals interested in measuring players' attributes without testing them in the traditional ways identified in this review. This study finds that fewer ice hockey tests are conducted in female cohorts and that this trend should be reversed in future research so as to develop this research area and its practical application to female ice hockey athletes. Due to the large variability of testing procedures concerning agility and RSSA capacities, future research should also aim to determine which assessment is most highly related to in-game performance, despite the issues mentioned above, to validate and standardize an on-ice RSSA test useful for teams' coaches and researchers.

## 5. Conclusion

Considering ice hockey match requirements and protocols used in the literature, the systematic review highlights the widespread use of on-ice skating tests to assess ability to repeat intense efforts, agility, acceleration and speed on the ice. Since on-ice tests relate specifically to the actions of the ice hockey player, we believe that ice hockey stakeholders can benefit from this practical and useful inventory tool through the guidance of tests adapted to the characteristics of the athlete populations they work with. Despite the issues and constraints of athletes’ testing, there is a need to assess as close as possible to real game conditions, on the ice with a full protective equipment, by using various specific skating patterns. This review proposes relevant options and solutions for researchers and practitioners (i.e., ice hockey coaches, on-ice skills specialists, strength and conditioning coaches, athletic therapists) who aim to integrate on-ice testing with different populations and objectives. The increasing emphasis on age-related on-ice evaluation indicates that ice hockey is a late-developing sport, where assessment becomes more relevant at an advanced age of expertise. Most research designs do not yet consider the associations between on-ice testing and performance in real competition settings. Indeed, live-match data are also complicated to collect, whether from an ethical, methodological or technological perspective. This review also suggests a need and relevance for developing and validating tests that assess ice hockey-specific skills and decision-making abilities. Performance in ice hockey is multi-factorial and depends on physical attributes (e.g., acceleration capacity, speed, power) and individual technical abilities (e.g., skating technique, capacity to change direction efficiently and quickly, passing accuracy). However, the interaction between the expression of these components and the unpredictable context of intermittent team sports remains an area that has not yet been fully assessed and decrypted. Agility is a complex but fundamental quality to evolve at the highest levels, refers to information and decision-making, playing intelligence, the core elements which each athlete must develop during their sporting career and which are challenging to evaluate from an ecological and specific perspective.

## Data Availability

The raw data supporting the conclusions of this article will be made available by the authors, without undue reservation.

## References

[1] Brocherie F Girard O Millet GP. Updated analysis of changes in locomotor activities across periods in an international ice hockey game. Biol Sport. (2018) 35(3):261–7. 10.5114/biolsport.2018.77826 30449944 PMC6224850

[2] Neeld K. Preparing for the demands of professional hockey. Strength Cond J. (2018) 40(2):1–16. 10.1519/ssc.0000000000000374

[3] Montgomery DL. Physiology of ice hockey. Sports Med. (1988) 5(2):99–126. 10.2165/00007256-198805020-00003 3281210

[4] Cox MH Miles DS Verde TJ Rhodes EC. Applied physiology of ice hockey. Sports Med. (1995) 19(3):184–201. 10.2165/00007256-199519030-00004 7784758

[5] Bracko MR Fellingham GW. Comparison of physical performance characteristics of female and male ice hockey players. Pediatr Exerc Sci. (2001) 13(1):26–34. 10.1123/pes.13.1.26

[6] Tarter BC Kirisci L Tarter RE Weatherbee S Jamnik V McGuire EJ Use of aggregate fitness indicators to predict transition into the national hockey league. J Strength Cond Res. (2009) 23(6):1828–32. 10.1519/JSC.0b013e3181b4372b 19675476

[7] Gannon EA Higham DG Gardner BW Nan N Zhao J Bisson LJ. Changes in neuromuscular status across a season of professional men’s ice hockey. J Strength Cond Res. (2021) 35(5):1338–44. 10.1519/JSC.0000000000004001 33651739

[8] Nightingale SC. A strength and conditioning approach for ice hockey. Strength Cond J. (2014) 36(6):28–36. 10.1519/ssc.0000000000000107

[9] Daehlin TE Haugen OC Haugerud S Hollan I Raastad T Ronnestad BR. Improvement of ice hockey players’ on-ice sprint with combined plyometric and strength training. Int J Sports Physiol Perform. (2017) 12(7):893–900. 10.1123/ijspp.2016-0262 27918670

[10] Lagrange S Ferland PM Leone M Comtois AS. Contrast training generates post-activation potentiation and improves repeated sprint ability in elite ice hockey players. Int J Exerc Sci. (2020) 13(6):183–96. 32148640 10.70252/ONUV8208 PMC7039519

[11] Lemoyne J Brunelle JF Huard Pelletier V Glaude-Roy J Martini G. Talent identification in elite adolescent ice hockey players: the discriminant capacity of fitness tests, skating performance and psychological characteristics. Sports. (2022) 10(4):58. 10.3390/sports10040058 35447868 PMC9026156

[12] Vigh-Larsen JF Beck JH Daasbjerg A Knudsen CB Kvorning T Overgaard K Fitness characteristics of elite and subelite male ice hockey players: a cross-sectional study. J Strength Cond Res. (2019) 33(9):2352–60. 10.1519/JSC.0000000000003285 31343551

[13] Martini G Brunelle JF Trudeau F Lemoyne J. Measuring ice hockey skills in a repeated measures testing context: the effects of fatigue on skating efficiency, passing, agility, and shooting. Sport J. (2018) 21:1–16.

[14] Perez J Guilhem G Brocherie F. Reliability of the force-velocity-power variables during ice hockey sprint acceleration. Sports Biomech. (2022b) 21(1):56–70. 10.1080/14763141.2019.1648541 31464169

[15] Douglas AS Rotondi MA Baker J Jamnik VK Macpherson AK. A comparison of on-ice external load measures between subelite and elite female ice hockey players. J Strength Cond Res. (2022) 36(7):1978–83. 10.1519/JSC.0000000000003771 32796414

[16] Gamble ASD Bigg JL Pignanelli C Nyman DLE Burr JF Spriet LL. Reliability and validity of an indoor local positioning system for measuring external load in ice hockey players. Eur J Sport Sci. (2023) 23(3):311–8. 10.1080/17461391.2022.2032371 35062856

[17] Cordingley DM Sirant L MacDonald PB Leiter JR. Three-year longitudinal fitness tracking in top-level competitive youth ice hockey players. J Strength Cond Res. (2019) 33(11):2909–12. 10.1519/JSC.0000000000003379 31644516

[18] Martini G Brunelle JF Lalande V Lemoyne J. Elite adolescent ice hockey players: analyzing associations between anthropometry, fitness, and on-ice performance. Int J Environ Res Public Health. (2022) 19(15):8952. 10.3390/ijerph19158952 35897327 PMC9330307

[19] Worner T Thorborg K Eek F. Five-second squeeze testing in 333 professional and semiprofessional male ice hockey players: how are hip and groin symptoms, strength, and sporting function related? Orthop J Sports Med. (2019) 7(2):2325967119825858. 10.1177/2325967119825858 30815497 PMC6383089

[20] Oliveras R Bizzini M Brunner R Maffiuletti NA. Field-based evaluation of hip adductor and abductor strength in professional male ice hockey players: reference values and influencing factors. Phys Ther Sport. (2020) 43:204–9. 10.1016/j.ptsp.2020.03.006 32222647

[21] Delisle-Houde P Reid RER Insogna JA Chiarlitti NA Andersen RE. Seasonal changes in physiological responses and body composition during a competitive season in male and female elite collegiate ice hockey players. J Strength Cond Res. (2019b) 33(8):2162–9. 10.1519/JSC.0000000000002338 31344012

[22] Nightingale SC Miller S Turner A. The usefulness and reliability of fitness testing protocols for ice hockey players: a literature review. J Strength Cond Res. (2013) 27(6):1742–8. 10.1519/JSC.0b013e3182736948 22996029

[23] Cohen JN Thompson KMA Jamnik VK Gledhill N Burr JF. Relationship of fitness combine results and national hockey league performance: a 25-year analysis. Int J Sports Physiol Perform. (2022) 17(6):908–16. 10.1123/ijspp.2021-0317 35245896

[24] Vescovi JD Murray TM Fiala KA VanHeest JL. Off-ice performance and draft status of elite ice hockey players. Int J Sports Physiol Perform. (2006) 1(3):207–21. 10.1123/ijspp.1.3.207 19116435

[25] Ferguson RJ Marcotte G Montpetit RR. A maximal oxygen uptake test during ice skating. Med Sci Sports. (1969) 1:207–11. 10.1249/00005768-196912000-00007

[26] Hermiston RT Gratto J Teno T. Three hockey skills tests as predictors of hockey playing ability. Can J Appl Sport Sci. (1979) 4(1):95–7. 498410

[27] Reed A Hasen H Cotton C Gauthier R Jette M Thoden J Development and validation of an on-ice hockey fitness test. Can J Appl Sport Sci. (1979) 4:245.

[28] Bracko MR. On-ice performance characteristics of elite and non-elite women’s ice hockey players. J Strength Cond Res. (2001) 15(1):42–7. 11708705

[29] Conners RT Whitehead PN Dodds FT Schott KD Quick MC. Validation of the polar team pro system for sprint speed with ice hockey players. J Strength Cond Res. (2022) 36(12):3468–72. 10.1519/JSC.0000000000003784 32881841

[30] Thompson KMA Safadie A Ford J Burr JF. Off-ice resisted sprints best predict all-out skating performance in varsity hockey players. J Strength Cond Res. (2022) 36(9):2597–601. 10.1519/JSC.0000000000003861 33136771

[31] Bracko MR. Biomechanics powers ice hockey performance. Biomechanics. (2004) 9:47–53.

[32] Budarick AR Shell JR Robbins SMK Wu T Renaud PJ Pearsall DJ. Ice hockey skating sprints: run to glide mechanics of high caliber male and female athletes. Sports Biomech. (2020) 19(5):601–17. 10.1080/14763141.2018.1503323 30200818

[33] Robbins SM Renaud PJ Pearsall DJ. Principal component analysis identifies differences in ice hockey skating stride between high- and low-calibre players. Sports Biomech. (2021) 20(2):131–49. 10.1080/14763141.2018.1524510 30411998

[34] Bracko MR Fellingham GW Hall LT Fisher AG Cryer W. Performance skating characteristics of professional ice hockey forwards. Sports Med Train Rehabil. (1998) 8(3):251–63. 10.1080/15438629809512531

[35] Nadeau L Godbout P Richard J-F. Assessment of ice hockey performance in real-game conditions. Eur J Sport Sci. (2008) 8(6):379–88. 10.1080/17461390802284456

[36] Van Iterson EH Fitzgerald JS Dietz CC Snyder EM Peterson BJ. Reliability of triaxial accelerometry for measuring load in men’s collegiate ice hockey. J Strength Cond Res. (2017) 31(5):1305–12. 10.1519/JSC.0000000000001611 27548782

[37] Douglas AS Kennedy CR. Tracking in-match movement demands using local positioning system in world-class men’s ice hockey. J Strength Cond Res. (2020) 34(3):639–46. 10.1519/JSC.0000000000003414 31855927

[38] Perez J Brocherie F Couturier A Guilhem G. International matches elicit stable mechanical workload in high-level female ice hockey. Biol Sport. (2022a) 39(4):857–64. 10.5114/biolsport.2022.109455 36247938 PMC9536379

[39] Peterson BJ Fitzgerald JS Dietz CC Ziegler KS Baker SE Snyder EM. Off-ice anaerobic power does not predict on-ice repeated shift performance in hockey. J Strength Cond Res. (2016) 30(9):2375–81. 10.1519/JSC.0000000000001341 26808844

[40] Chiarlitti NA Crozier M Insogna JA Reid RER Delisle-Houde P. Longitudinal physiological and fitness evaluations in elite ice hockey: a systematic review. J Strength Cond Res. (2021) 35(10):2963–79. 10.1519/JSC.0000000000004115 34387221

[41] Stastny P Musalek M Roczniok R Cleather D Novak D Vagner M. Testing distance characteristics and reference values for ice-hockey straight sprint speed and acceleration. A systematic review and meta-analyses. Biol Sport. (2023) 40:899–918. 10.5114/biolsport.2023.122479 37398950 PMC10286618

[42] Huard Pelletier V Glaude-Roy J Daigle AP Brunelle JF Bissonnette A Lemoyne J. Associations between testing and game performance in ice hockey: a scoping review. Sports. (2021) 9(9):117. 10.3390/sports9090117 34564322 PMC8473052

[43] Baumeister RF. Writing a literature review. In: Prinstein MJ, editors. The portable mentor. New York, NY, US: Springer Science Business Media (2013). p. 119–32.

[44] Page MJ McKenzie JE Bossuyt PM Boutron I Hoffmann TC Mulrow CD The PRISMA 2020 statement: an updated guideline for reporting systematic reviews. Syst Rev. (2021) 10(1):89. 10.1186/s13643-021-01626-4 33781348 PMC8008539

[45] Triplett AN Ebbing AC Green MR Connolly CP Carrier DP Pivarnik JM. Changes in collegiate ice hockey player anthropometrics and aerobic fitness over 3 decades. Appl Physiol Nutr Metab. (2018) 43(9):950–5. 10.1139/apnm-2017-0789 29629563

[46] PubMed. PubMed publications (ice hockey and test*) (2023). Available at: https://pubmed.ncbi.nlm.nih.gov/?term=ice%20hockey%20and%20test*%20&timeline=expanded (Accessed July 22, 2023).

[47] Methley AM Campbell S Chew-Graham C McNally R Cheraghi-Sohi S. PICO, PICOS and SPIDER: a comparison study of specificity and sensitivity in three search tools for qualitative systematic reviews. BMC Health Serv Res. (2014) 14:579. 10.1186/s12913-014-0579-0 25413154 PMC4310146

[48] Vigh-Larsen JF Mohr M. The physiology of ice hockey performance: an update. Scand J Med Sci Sports. (2022) 00:1–14. 10.1111/sms.14284 36517860

[49] Nightingale SC. Ice hockey: the validity and reliability of a novel on-ice test for ice hockey players. Professional Strength Cond. (2013) 31:15–8.

[50] Novak D Lipinska P Roczniok R Spieszny M Stastny P. Off-ice agility provide motor transfer to on-ice skating performance and agility in adolescent ice hockey players. J Sports Sci Med. (2019) 18(4):680–94. 31827353 PMC6873137

[51] Girard O Mendez-Villanueva A Bishop D. Repeated-sprint ability - part I: factors contributing to fatigue. Sports Med. (2011) 41(8):673–94. 10.2165/11590550-000000000-00000 21780851

[52] Stanula A Gupta S Baron J Bieniec A Tomik R Gabrys T A comparative study of two-minute versus three-minute passive recovery on sprint skating performance of ice hockey forwards and defensemen. Int J Environ Res Public Health. (2021) 18(24):10591. 10.3390/ijerph182413029 34948639 PMC8701228

[53] International Ice Hockey Federation. “Youth Olympic Games National Skills Challenge Operations Manual” (2020).

[54] Hockey Canada. *National Skills Standards and Testing Program Handbook*. Available at: https://cdn.hockeycanada.ca/hockey-canada/Hockey-Programs/Players/Skills-Testing/Downloads/nsst_handbook_e.pdf (Accessed) (2022).

[55] Balyi I. *Long-term Planning of Athlete Development, Multiple Periodization, Modeling and Normative Data* (1999).

[56] Allisse M Bui HT Desjardins P Léger L Comtois A-S Leone M. Assessment of on-ice oxygen cost of skating performance in elite youth ice hockey players. J Strength Cond Res. (2021) 35(12):3466–73. 10.1519/JSC.0000000000003324 31809459

[57] Allisse M Bui HT Léger L Comtois A-S Leone M. Updating the skating multistage aerobic test and correction for V˙O_2_max prediction using a new skating economy index in elite youth ice hockey players. J Strength Cond Res. (2020) 34(11):3182–9. 10.1519/jsc.0000000000002602 33105369

[58] Allisse M Sercia P Comtois A-S Leone M. Morphological, physiological and skating performance profiles of male age-group elite ice hockey players. J Hum Kinet. (2017) 58(1):87–97. 10.1515/hukin-2017-0085 28828080 PMC5548157

[59] Baron J Gupta S Bieniec A Klich G Gabrys T Swinarew AS Effect of rest period duration between sets of repeated sprint skating ability test on the skating ability of ice hockey players. Int J Environ Res Public Health. (2021) 18(20):10591. 10.3390/ijerph182010591 34682336 PMC8536092

[60] Behm DG Wahl MJ Button DC Power KE Anderson KG. Relationship between hockey skating speed and selected performance measures. J Strength Cond Res. (2005) 19(2):326–31. 10.1519/R-14043.1 15903370

[61] Besson C Buchheit M Praz M Dériaz O Millet GP. Cardiorespiratory responses to the 30-15 intermittent ice test. Int J Sports Physiol Perform. (2013) 8(2):173–80. 10.1123/ijspp.8.2.173 22904118

[62] Boland M Delude K Miele EM. Relationship between physiological off-ice testing, on-ice skating, and game performance in division I female ice hockey players. J Strength Cond Res. (2019) 33(6):1619–28. 10.1519/JSC.0000000000002265 29016475

[63] Bond CW Bennett TW Noonan BC. Evaluation of skating top speed, acceleration, and multiple repeated sprint speed ice hockey performance tests. J Strength Cond Res. (2018) 32(8):2273–83. 10.1519/JSC.0000000000002644 29878985

[64] Boucher VG Parent A-A Miron FS-J Leone M Comtois AS. Comparison between power off-ice test and performance on-ice anaerobic testing. J Strength Cond Res. (2020) 34(12):3498–505. 10.1519/JSC.0000000000002336 29210955

[65] Bracko MR George JD. Prediction of ice skating performance with off-ice testing in women’s ice hockey players. J Strength Cond Res. (2001) 15(1):116–22. 11708693

[66] Brocherie F Perez J Guilhem G. Effects of a 14-day high-intensity shock microcycle in high-level ice hockey players’ fitness. J Strength Cond Res. (2022) 36(8):2247–52. 10.1519/JSC.0000000000003769 32826829

[67] Buchheit M Lefebvre B Laursen PB Ahmaidi S. Reliability, usefulness, and validity of the 30-15 intermittent ice test in young elite ice hockey players. J Strength Cond Res. (2011) 25(5):1457–64. 10.1519/JSC.0b013e3181d686b7 21522077

[68] Buckeridge E LeVangie MC Stetter B Nigg SR Nigg BM. An on-ice measurement approach to analyse the biomechanics of ice hockey skating. PLoS One. (2015) 10(5):e0127324. 10.1371/journal.pone.0127324 25973775 PMC4431820

[69] Byrkjedal PT Bjørnsen T Luteberget LS Lindberg K Ivarsson A Haukali E Association between physical performance tests and external load during scrimmages in highly trained youth ice hockey players. Int J Sports Physiol Perform. (2022) 18(1):47–54. 10.1123/ijspp.2022-0225 36470253

[70] Carey DG Drake MM Pliego GJ Raymond RL. Do hockey players need aerobic fitness? Relation between V˙O_2_max and fatigue during high-intensity intermittent ice skating. J Strength Cond Res. (2007) 21(3):963–6. 10.1519/R-18881.1 17685680

[71] Czeck MA Roelofs EJ Dietz C Bosch TA Dengel DR. Body composition and on-ice skate times for national collegiate athletic association division I collegiate male and female ice hockey athletes. J Strength Cond Res. (2022) 36(1):187–92. 10.1519/JSC.0000000000004175 34941612

[72] Daigle A-P Bélanger S Brunelle J-F Lemoyne J. Functional performance tests, on-ice testing and game performance in elite junior ice hockey players. J Hum Kinet. (2022) 83(1):245–56. 10.2478/hukin-2022-000076 36157962 PMC9465767

[73] Delisle-Houde P Chiarlitti NA Reid RE Andersen RE. Predicting on-ice skating using laboratory-and field-based assessments in college ice hockey players. Int J Sports Physiol Perform. (2019a) 14(9):1184–9. 10.1123/ijspp.2018-0708 30840516

[74] Durocher JJ Guisfredi AJ Leetun DT Carter JR. Comparison of on-ice and off-ice graded exercise testing in collegiate hockey players. Appl Physiol Nutr Metab. (2010) 35(1):35–9. 10.1139/H09-129 20130664

[75] Durocher JJ Jensen DD Arredondo AG Leetun DT Carter JR. Gender differences in hockey players during on-ice graded exercise. J Strength Cond Res. (2008a) 22(4):1327–31. 10.1519/JSC.0b013e31816eb4c1 18545171

[76] Durocher JJ Leetun DT Carter JR. Sport-specific assessment of lactate threshold and aerobic capacity throughout a collegiate hockey season. Appl Physiol Nutr Metab. (2008b) 33(6):1165–71. 10.1139/H08-107 19088774

[77] Eliason PH McKay CD Meeuwisse WH Hagel BE Nadeau L Emery CA. History of previous concussion and sports-specific skills in youth ice hockey players. J Phys Educ Sport. (2020) 20(3):2174–81. 10.7752/jpes.2020.s3292

[78] Farlinger CM Fowles JR. The effect of sequence of skating-specific training on skating performance. Int J Sports Physiol Perform. (2008) 3(2):185–98. 10.1123/ijspp.3.2.185 19208927

[79] Farlinger CM Kruisselbrink LD Fowles JR. Relationships to skating performance in competitive hockey players. J Strength Cond Res. (2007) 21(3):915–22. 10.1519/R-19155.1 17685681

[80] Federolf P Redmond A. Does skate sharpening affect individual skating performance in an agility course in ice hockey? Sports Eng. (2010) 13:39–46. 10.1007/s12283-010-0050-3

[81] Federolf P Nigg B. Skating performance in ice hockey when using a flared skate blade design. Cold Reg Sci Technol. (2012) 70:12–8. 10.1016/j.coldregions.2011.08.009

[82] Ferland PM Marcotte-L’Heureux V Roy P Carey VD Charron J Lagrange S Maximal oxygen consumption requirements in professional North American ice hockey. J Strength Cond Res. (2021) 35(6):1586–92. 10.1519/JSC.0000000000003966 33555827

[83] Geithner CA. Predicting performance in women’s ice hockey. In: Duncan M Lyons M, editors. Advances in strength and conditioning research. New York, NY: Nova Science Publishers (2009). p. 51–63.

[84] Geithner CA Lee AM Bracko MR. Physical and performance differences among forwards, defensemen, and goalies in elite women’s ice hockey. J Strength Cond Res. (2006) 20(3):500–5. 10.1519/17375.1 16977704

[85] Gilenstam KM Thorsen K Henriksson-Larsén KB. Physiological correlates of skating performance in women’s and men’s ice hockey. J Strength Cond Res. (2011) 25(8):2133–42. 10.1519/JSC.0b013e3181ecd072 21785292

[86] Girdauskas G Kazakevičius R. Optimization of technical training of ice-hockey players aged 8–17 years. Ugdymas. Kūno kultūra. Sportas. (2013) 2:19–26. 10.33607/bjshs.v2i89.155

[87] Gupta S Baron J Bieniec A Swinarew A Stanula A. Relationship between vertical jump tests and ice-skating performance in junior Polish ice hockey players. Biol Sport. (2022) 40(1):225–32. 10.5114/biolsport.2023.112972 36636195 PMC9806740

[88] Hajek F Keller M Taube W von Duvillard SP Bell JW Wagner H. Testing-specific skating performance in ice hockey. J Strength Cond Res. (2021) 35:S70–5. 10.1519/JSC.0000000000003475 32149873

[89] Haukali E Tjelta LI. Correlation between “off-ice” variables and skating performance among young male ice hockey players. Int J Appl Sports Sci. (2015) 27(1):26–32. 10.24985/ijass.2015.27.1.26

[90] Haukali E Tjelta LI. Relationship between off-season changes in power and in-season changes in skating speed in young ice hockey players. Int J Appl Sports Sci. (2016) 28(2):111–22. 10.24985/ijass.2016.28.2.111

[91] Henriksson T Vescovi JD Fjellman-Wiklund A Gilenstam K. Laboratory-and field-based testing as predictors of skating performance in competitive-level female ice hockey. Open Access J Sports Med. (2016) 7:81. 10.2147/OAJSM.S109124 27574474 PMC4994876

[92] Hůlka K Bělka J Cuberek R Schneider O. Reliability of specific on-ice repeated-sprint ability test for ice-hockey players. Acta Univ Palacki Olomuc Gymn. (2014) 44(2):69–75. 10.5507/ag.2014.007

[93] Jackson J Snydmiller G Game A Gervais P Bell G. Investigation of positional differences in fitness of male university ice hockey players and the frequency, time spent and heart rate of movement patterns during competition. Int J Kinesiol Sports Sci. (2017) 5(3):6–15. 10.7575/aiac.ijkss.v.5n.3p.6

[94] Janot JM Auner KA Emberts TM Kaatz RM Matteson KM Muller EA The effects of bungeeskate training on measures of on-ice acceleration and speed. Int J Sports Physiol Perform. (2013) 8(4):419–27. 10.1123/ijspp.8.4.419 23237915

[95] Janot JM Beltz NM Dalleck LD. Multiple off-ice performance variables predict on-ice skating performance in male and female division III ice hockey players. J Sports Sci Med. (2015) 14(3):522–9. 26336338 PMC4541115

[96] Kaartinen S Venojärvi M Lesch KJ Tikkanen H Vartiainen P Stenroth L. Lower limb muscle activation patterns in ice-hockey skating and associations with skating speed. Sports Biomech. (2021):1–16. 10.1080/14763141.2021.2014551 [Epub ahead of print]. 34930101

[97] Kinnunen J-V Piitulainen H Piirainen JM. Neuromuscular adaptations to short-term high-intensity interval training in female ice-hockey players. J Strength Cond Res. (2019) 33(2):479–85. 10.1519/JSC.0000000000001881 28277422

[98] Knechta M Čillík I Zháněl J. Influence of plyometric training on the level of speed ability with changes of direction in ice hockey. Stud Sport. (2021) 15(1):17–25. 10.5817/StS2021-1-2

[99] Knechta M. Impact of explosive strength of lower limbs on skating and running speed on a 10 m distance in 14–15 years old ice hockey players: a recent study. Humanit Soc Sci. (2021) 1:30–7. 10.9734/bpi/sthss/v1/9029D

[100] Krause DA Smith AM Holmes LC Klebe CR Lee JB Lundquist KM Relationship of off-ice and on-ice performance measures in high school male hockey players. J Strength Cond Res. (2012) 26(5):1423–30. 10.1519/JSC.0b013e318251072d 22395275

[101] Lamoureux NR Tomkinson GR Peterson BJ Fitzgerald JS. Relationship between skating economy and performance during a repeated-shift test in elite and subelite ice hockey players. J Strength Cond Res. (2018) 32(4):1109–13. 10.1519/JSC.0000000000002418 29324580

[102] Lau S Berg K Latin RW Noble J. Comparison of active and passive recovery of blood lactate and subsequent performance of repeated work bouts in ice hockey players. J Strength Cond Res. (2001) 15(3):367–71. 10.1519/1533-4287(2001)015<0367:COAAPR>2.0.CO;2 11710667

[103] Lee C Lee S Yoo J. The effect of a complex training program on skating abilities in ice hockey players. J Phys Ther Sci. (2014) 26(4):533–7. 10.1589/jpts.26.533 24764628 PMC3996416

[104] Legerlotz K Kittelmann J Dietzel M Wolfarth B Bohlke N. Ice hockey-specific repeated shuttle sprint test performed on ice should not be replaced by off-ice testing. J Strength Cond Res. (2022) 36(4):1071–6. 10.1519/JSC.0000000000003576 32218060

[105] Leone M Léger LA Larivière G Comtois AS. An on-ice aerobic maximal multistage shuttle skate test for elite adolescent hockey players. Int J Sports Med. (2007) 28(10):823–8. 10.1055/s-2007-964986 17534782

[106] Lignell E Fransson D Krustrup P Mohr M. Analysis of high-intensity skating in top-class ice hockey match-play in relation to training status and muscle damage. J Strength Cond Res. (2018) 32(5):1303–10. 10.1519/JSC.0000000000001999 28557852

[107] Lowery MR Tomkinson GR Peterson BJ Fitzgerald JS. The relationship between ventilatory threshold and repeated-sprint ability in competitive male ice hockey players. J Exerc Sci Fit. (2018) 16(1):32–6. 10.1016/j.jesf.2018.03.003 30662490 PMC6323167

[108] Madden RF Erdman KA Shearer J Spriet LL Ferber R Kolstad AT Effects of caffeine on exertion, skill performance, and physicality in ice hockey. Int J Sports Physiol Perform. (2019) 14(10):1422–9. 10.1123/ijspp.2019-0130 30958066

[109] Matthews MJ Comfort P Crebin R. Complex training in ice hockey: the effects of a heavy resisted sprint on subsequent ice-hockey sprint performance. J Strength Cond Res. (2010) 24(11):2883–7. 10.1519/JSC.0b013e3181e7253c 20940636

[110] Naimo M De Souza E Wilson J Carpenter A Gilchrist P Lowery R High-intensity interval training has positive effects on performance in ice hockey players. Int J Sports Med. (2015) 36(01):61–6. 10.1055/s-0034-1382054 25329432

[111] Nigg CR Gessner A Nigg C Giurgiu M Neumann R. Demographische, physiologische, psychologische und on-ice leistungsindikatoren sagen die plus/minus-statistik von freizeit-eishockeyspielern über eine saison voraus. Ger J Exerc Sport Res. (2020) 50:463–9. 10.1007/s12662-020-00679-2

[112] Nobes K Montgomery D Pearsall D Turcotte R Lefebvre R Whittom F. A comparison of skating economy on-ice and on the skating treadmill. Can J Appl Physiol. (2003) 28(1):1–11. 10.1139/h03-001 12649528

[113] Novak D Tomasek A Lipinska P Stastny P. The specificity of motor learning tasks determines the kind of skating skill development in older school-age children. Sports. (2020) 8(9):126. 10.3390/sports8090126 32937807 PMC7552761

[114] Opáth L. Powerskating as a method of skating development in category older students and youth team. J Phys Educ Sport Health. (2015) 4(2):17–21.

[115] Paľov R. Influence of the time of the day and chronotype on speed abilities in junior team ice hockey players. SportLogia. (2014) 10(2):122–8. 10.5550/sgia.141002.en.009P

[116] Perez J Guilhem G Brocherie F. Ice hockey forward skating force-velocity profiling using single unloaded vs. multiple loaded methods. J Strength Cond Res. (2021a) 36(11):3229–33. 10.1519/jsc.0000000000004078 34175878

[117] Perez J Guilhem G Hager R Brocherie F. Mechanical determinants of forward skating sprint inferred from off-and on-ice force-velocity evaluations in elite female ice hockey players. Eur J Sport Sci. (2021b) 21(2):192–203. 10.1080/17461391.2020.1751304 32241241

[118] Peterson BJ Fitzgerald JS Dietz CC Ziegler KS Ingraham SJ Baker SE Division I hockey players generate more power than division III players during on-and off-ice performance tests. J Strength Cond Res. (2015b) 29(5):1191–6. 10.1519/JSC.0000000000000754 25436625

[119] Peterson BJ Fitzgerald JS Dietz CC Ziegler KS Ingraham SJ Baker SE Aerobic capacity is associated with improved repeated shift performance in hockey. J Strength Cond Res. (2015a) 29(6):1465–72. 10.1519/JSC.0000000000000786 25756322

[120] Petrella NJ Montelpare WJ Nystrom M Plyley M Faught BE. Validation of the FAST skating protocol to predict aerobic power in ice hockey players. Appl Physiol Nutr Metab. (2007) 32(4):693–700. 10.1139/H07-057 17622284

[121] Peyer KL Pivarnik JM Eisenmann JC Vorkapich M. Physiological characteristics of national collegiate athletic association division I ice hockey players and their relation to game performance. J Strength Cond Res. (2011) 25(5):1183–92. 10.1519/JSC.0b013e318217650a 21478763

[122] Potteiger JA Smith DL Maier ML Foster TS. Relationship between body composition, leg strength, anaerobic power, and on-ice skating performance in division I men’s hockey athletes. J Strength Cond Res. (2010) 24(7):1755–62. 10.1519/JSC.0b013e3181e06cfb 20543730

[123] Power A Faught B Przysucha E McPherson M Montelpare W. Establishing the test–retest reliability and concurrent validity for the repeat ice skating test (RIST) in adolescent male ice hockey players. Meas Phys Educ Exerc Sci. (2012) 16(1):69–80. 10.1080/1091367X.2012.639618

[124] Rago V Muschinsky A Deylami K Vigh-Larsen JF Mohr M. Game demands of a professional ice hockey team with special emphasis on fatigue development and playing position. J Hum Kinet. (2022) 84:195–205. 10.2478/hukin-2022-000078 36457463 PMC9679183

[125] Roczniok R Maszczyk A Czuba M Stanula A Pietraszewski P Gabryś T. The predictive value of on-ice special tests in relation to various indexes of aerobic and anaerobic capacity in ice hockey players. Hum Mov. (2012) 13(1):28–32. 10.2478/v10038-012-0001-x

[126] Roczniok R Maszczyk A Stanula A Czuba M Pietraszewski P Kantyka J Physiological and physical profiles and on-ice performance approach to predict talent in male youth ice hockey players during draft to hockey team. Isokinet Exerc Sci. (2013) 21(2):121–7. 10.3233/ies-130487

[127] Roczniok R Stanula A Gabryś T Szmatlan-Gabryś U Gołaś A Stastny P. Physical fitness and performance of polish ice-hockey players competing at different sports levels. J Hum Kinet. (2016a) 51(1):201–8. 10.1515/hukin-2015-0165 28149383 PMC5260545

[128] Roczniok R Stanula A Maszczyk A Mostowik A Kowalczyk M Fidos-Czuba O Physiological, physical and on-ice performance criteria for selection of elite ice hockey teams. Biol Sport. (2016b) 33(1):43–8. 10.5604/20831862.1180175 26985133 PMC4786585

[129] Rønnestad BR Haugen OC Dæhlin TE. Superior on-ice performance after short-interval vs. long-interval training in well-trained adolescent ice hockey players. J Strength Cond Res. (2021) 35:S76–80. 10.1519/jsc.0000000000004113 34334774

[130] Runner AR Lehnhard RA Butterfield SA Tu S O'Neill T. Predictors of speed using off-ice measures of college hockey players. J Strength Cond Res. (2016) 30(6):1626–32. 10.1519/jsc.0000000000000911 25719922

[131] Schwesig R Hermassi S Edelmann S Thorhauer U Schulze S Fieseler G Relationship between ice hockey-specific complex test and maximal strength, aerobic capacity and postural regulation in professional players. J Sports Med Phys Fit. (2017) 57(11):1415–23. 10.23736/s0022-4707.17.07020-7 28139111

[132] Secomb JL Dascombe BJ Nimphius S. Importance of joint angle-specific hip strength for skating performance in semiprofessional ice hockey athletes. J Strength Cond Res. (2021) 35(9):2599–603. 10.1519/JSC.0000000000004087 34431485

[133] Shell JR Robbins SM Dixon PC Renaud PJ Turcotte RA Wu T Skating start propulsion: three-dimensional kinematic analysis of elite male and female ice hockey players. Sports Biomech. (2017) 16(3):313–24. 10.1080/14763141.2017.1306095 28534433

[134] Skowronek T Socha T Roczniok R Socha S. The predictive value of various anaerobic capacity indices in relation to specific on-ice performance tests in ice hockey players. Life Sci J. (2013) 10(4):2228–832.

[135] Slavicek T Stastny P Roczniok R Musalek M. Lower limb skeletal robustness determines the change of directional speed performance in youth ice hockey. J Hum Kinet. (2022) 85:75–85. 10.2478/hukin-2022-0111 36643831 PMC9808801

[136] Smith AM Krause DA Stuart MJ Montelpare WJ Sorenson MC Link AA Skating crossovers on a motorized flywheel: a preliminary experimental design to test effect on speed and on crossovers. J Strength Cond Res. (2013) 27(12):3412–8. 10.1519/JSC.0b013e3182915f37 23539081

[137] Stanula A Roczniok R Maszczyk A Pietraszewski P Zajac A. The role of aerobic capacity in high-intensity intermittent efforts in ice-hockey. Biol Sport. (2014) 31(3):193–9. 10.5604/20831862.1111437 25177097 PMC4135063

[138] Steeves D Campagna P. The relationship between maximal aerobic power and recovery in elite ice hockey players during a simulated game. J Strength Cond Res. (2019) 33(9):2503–12. 10.1519/JSC.0000000000002506 29461415

[139] Stenroth L Vartiainen P Karjalainen PA. Force-velocity profiling in ice hockey skating: reliability and validity of a simple, low-cost field method. Sports Biomech. (2020) 22(7):1–16. 10.1080/14763141.2020.1770321 32546104

[140] Stetter BJ Buckeridge E Nigg SR Sell S Stein T. Towards a wearable monitoring tool for in-field ice hockey skating performance analysis. Eur J Sport Sci. (2019) 19(7):893–901. 10.1080/17461391.2018.1563634 30606093

[141] Vigh-Larsen JF Ermidis G Rago V Randers MB Fransson D Nielsen JL Muscle metabolism and fatigue during simulated ice hockey match-play in elite players. Med Sci Sports Exercise. (2020a) 52(10):2162–71. 10.1249/MSS.0000000000002370 32496739

[142] Vigh-Larsen JF Haverinen MT Panduro J Ermidis G Andersen TB Overgaard K On-ice and off-ice fitness profiles of elite and U20 male ice hockey players of two different national standards. J Strength Cond Res. (2020b) 34(12):3369–76. 10.1519/JSC.0000000000003836 33009345

[143] Wagner H Abplanalp M von Duvillard SP Bell JW Taube W Keller M. The relationship between on-ice and off-ice performance in elite male adolescent ice hockey players—an observation study. Appl Sci. (2021) 11(6):2724. 10.3390/app11062724

[144] Williams M Grau S. Physical performance and the relationship to game performance in elite adolescent ice hockey. Int J Strength Conf. (2020) 1(1):1–11. 10.47206/iuscaj.v1i1.3

[145] Wilson K Jackson J Snydmiller G Bell G. Development and reliability of a 7 (15 m repeated on-ice sprint test for female ice hockey players. Int J Exerc Sci. (2021) 14(6):666–76. 34567374 10.70252/RPCO2660 PMC8439675

[146] Ransdell LB Murray TM Gao Y. Off-ice fitness of elite female ice hockey players by team success, age, and player position. J Strength Cond Res. (2013) 27(4):875–84. 10.1519/JSC.0b013e3182651fd2 22739329

[147] Hockey Canada. “Plan de Hockey Canada pour le développement à long terme du joueur”.) (2013).

[148] Higgs C Way R Harber V Jurbala P Balyi I. “Développement à long terme par le sport et l'activité physique”, C.S. Institute, 3rd edition (2019).

[149] Lloyd RS Cronin JB Faigenbaum AD Haff GG Howard R Kraemer WJ National strength and conditioning association position statement on long-term athletic development. J Strength Cond Res. (2016) 30(6):1491–509. 10.1519/JSC.0000000000001387 26933920

[150] Laakso L. Critical evaluation of the physiological adaptations to repeated-sprint training: implications for training recommendations and repeated-sprint ability of well-trained field-based team-sport athletes. J Aust Strength Cond Res. (2020) 28(4):75–81.

[151] Schwesig R Laudner KG Delank K-S Brill R Schulze S. Relationship between ice hockey-specific complex test (IHCT) and match performance. Appl Sci. (2021) 11(7):3080. 10.3390/app11073080

[152] Heller J Vodicka P Janek M. Anaerobic performance in 30s wingate test as one of the possible criteria for selection Czech hockey players into national hockey league. Phys Act Rev. (2019) 7:57–62. 10.16926/par.2019.07.07

[153] Luteberget LS Gilgien M. Validation methods for global and local positioning-based athlete monitoring systems in team sports: a scoping review. BMJ Open Sport Exerc Med. (2020) 6(1):e000794. 10.1136/bmjsem-2020-000794 33062300 PMC7520549

[154] Vats K Walters P Fani M Clausi DA Zelek JS. Player tracking and identification in ice hockey. Expert Syst Appl. (2023) 213:119250. 10.1016/j.eswa.2022.119250

